# Comparative Multifractal Analysis of Dynamic Infrared Thermograms and X-Ray Mammograms Enlightens Changes in the Environment of Malignant Tumors

**DOI:** 10.3389/fphys.2016.00336

**Published:** 2016-08-09

**Authors:** Evgeniya Gerasimova-Chechkina, Brian Toner, Zach Marin, Benjamin Audit, Stephane G. Roux, Francoise Argoul, Andre Khalil, Olga Gileva, Oleg Naimark, Alain Arneodo

**Affiliations:** ^1^Laboratory of Physical Foundation of Strength, Institute of Continuous Media Mechanics UB RASPerm, Russia; ^2^CompuMAINE Laboratory, Department of Mathematics and Statistics, University of MaineOrono, ME, USA; ^3^Université Lyon, Ecole Normale Supérieure de Lyon, Université Claude Bernard Lyon 1, Centre National de la Recherche Scientifique, Laboratoire de PhysiqueLyon, France; ^4^Laboratoire Ondes et Matière d'Aquitaine, Centre National de la Recherche Scientifique, Université de Bordeaux, UMR 5798Talence, France; ^5^Department of Therapeutic and Propedeutic Dentistry, Perm State Medical UniversityPerm, Russia

**Keywords:** breast cancer, dynamic IR thermograms, X-ray mammograms, multifractal analysis, wavelet transform modulus maxima method

## Abstract

There is growing evidence that the microenvironment surrounding a tumor plays a special role in cancer development and cancer therapeutic resistance. Tumors arise from the dysregulation and alteration of both the malignant cells and their environment. By providing tumor-repressing signals, the microenvironment can impose and sustain normal tissue architecture. Once tissue homeostasis is lost, the altered microenvironment can create a niche favoring the tumorigenic transformation process. A major challenge in early breast cancer diagnosis is thus to show that these physiological and architectural alterations can be detected with currently used screening techniques. In a recent study, we used a 1D wavelet-based multi-scale method to analyze breast skin temperature temporal fluctuations collected with an IR thermography camera in patients with breast cancer. This study reveals that the multifractal complexity of temperature fluctuations superimposed on cardiogenic and vasomotor perfusion oscillations observed in healthy breasts is lost in malignant tumor foci in cancerous breasts. Here we use a 2D wavelet-based multifractal method to analyze the spatial fluctuations of breast density in the X-ray mammograms of the same panel of patients. As compared to the long-range correlations and anti-correlations in roughness fluctuations, respectively observed in dense and fatty breast areas, some significant change in the nature of breast density fluctuations with some clear loss of correlations is detected in the neighborhood of malignant tumors. This attests to some architectural disorganization that may deeply affect heat transfer and related thermomechanics in breast tissues, corroborating the change to homogeneous monofractal temperature fluctuations recorded in cancerous breasts with the IR camera. These results open new perspectives in computer-aided methods to assist in early breast cancer diagnosis.

## 1. Introduction

The past 30 years have seen the emergence in cancer biology of the concepts of local microenvironments and stem cell niches not only as key regulators of tissue specificity, homeostasis maintenance and tumor control, but also as active players in cancer initiation and progression to metastasis (Bissell et al., 2005; Bissell and Hines, 2011; Maguer-Satta, 2011; Lu et al., 2012). Important progress was made in the understanding of the permanent dialogue that exists between stem cells and their microenvironment (Fuchs et al., 2004; Li and Li, 2006; Moore and Lemischka, 2006; Morrison and Spradling, 2008). The role of the niche in normal tissues is critical in regulating asymmetric cell division and stem cell fate (Fuchs et al., 2004; Morrison and Kimble, 2006; Ho and Wagner, 2007; Marthiens et al., 2010). On the one hand, it participates to maintain stem cell quiescence until self-renewal divisions required to sustain the stem cell pool from exhaustion and alteration, and, when needed, to facilitate the proliferation necessary to respond to some physiological demand or injury (Morrison and Kimble, 2006; Morrison and Spradling, 2008; Trumpp et al., 2010). On the other hand, it contributes to drive cell differentiation processes during lineage specification and organ development (Ho and Wagner, 2007; Marthiens et al., 2010; Trumpp et al., 2010). Upon asymmetric division, cell commitment is distributed upon two daughter cells; one of them keeps stem cell properties while the other one is driven toward a more differentiated stage to respond, match, and adapt to the surrounding tissue (Marthiens et al., 2010). But an imbalance between stem cell activation and differentiation can lead to the generation of damaged stem cells by either overexpanding the stem cell pool or a failure in stem cell differentiation (Demicheli, 2001; Bissell et al., 2005; Bissell and Labarge, 2005; Flynn and Kaufman, 2007). In homeostatic conditions, the stromal environment of the niche, including fibroblast, vasculature and immune cells as well as interstitial extracellular matrix (ECM), can biochemically and biomechanically control the so-called cancer stem cells to maintain tissue architecture and integrity (Bissell and Hines, 2011; Maguer-Satta, 2011; Lu et al., 2012). This explains why many occult tumors can lie dormant or evolve very slowly (Demicheli, 2001; Bissell et al., 2005; Bissell and Labarge, 2005; Faraldo et al., 2005; Li and Neaves, 2006; Moore and Lemischka, 2006; Flynn and Kaufman, 2007; Tysnes and Bjerkvig, 2007). There is increasing evidence that the destabilization of tissue homeostasis originates from the alteration of the tumor microenvironment that not only affects the behavior of cancer stem cells, stroma and various epithelial cells, but also contributes to transform the niche into a tumorigenic microenvironment that further facilitates the process of oncogenic transformation, tissue invasion and metastasis evasion during cancer progression (Bissell et al., 2005; Lee and Herlyn, 2007; Rønnov-Jessen and Bissell, 2009; Bissell and Hines, 2011; Maguer-Satta, 2011; Lu et al., 2012). By favoring the survival and proliferation of cancer stem cells, this altered cancer promoting environment can maintain the quiescence of these cells and make them resistant to treatment, creating the possibility of regenerating a tumor (Demicheli, 2001; Hall et al., 2007; Besançon et al., 2009; Bissell and Hines, 2011; Maguer-Satta, 2011). The ongoing progress in understanding the fundamental role of cancer stem cells within their niche raises very challenging issues in cancer assessment, diagnosis and therapy. There are emerging strategies and recent clinical trials relevant to microenvironmental therapies (Hall et al., 2007; Besançon et al., 2009; Bissell and Hines, 2011; Maguer-Satta, 2011). In this work, we combine the analysis of dynamic IR thermograms and X-ray mammograms to show that changes in the environment of a malignant breast tumor can be detected with commonly used non invasive screening techniques.

Breast cancer is one of the major causes of death among women worldwide (Siegel et al., 2015). Clinical studies have demonstrated that survival is significantly improved if the breast anomalies are detected as early as possible (Lee, 2002). Despite some criticism of the use of screening techniques due to overdiagnosis (Jørgensen and Gøtzsche, 2009; Fenton et al., 2011), early detection remains the best strategy for improving prognosis and providing less invasive options for both specific diagnosis and treatment (Ganesan et al., 2013; Jalalian et al., 2013). In a preliminary study (Gerasimova et al., 2013, 2014), we showed that skin temperature dynamics recorded with an infrared (IR) camera displayed qualitative changes around malignant breast tumors. Using a wavelet-based time-frequency method, we demonstrated that temperature temporal fluctuations superimposed on the cardiogenic and vasomotor perfusion oscillations are not instrumental noise, but contain physiological information that can be exploited to anticipate the transition to malignancy. The observed drastic simplification from multifractal (continuous change of statistics across time-scales) to homogeneous monofractal (invariant statistics across time-scales) skin temperature fluctuations in malignant tumor foci, was hypothesized to be the signature of blood vessels and other tissues showing signs of aberration and architectural disintegration, likely affecting heat transfer and related thermomechanics inside the breast. The aim of the present study is to strengthen this interpretation by showing that this disorganization of the breast tissue architecture and vascular network in the environment of a malignant tumor can also efficiently be detected with X-ray mammography, the golden standard for breast cancer screening detection (Nass et al., 2001; Bronzino, 2006). Using a wavelet-based space-scale method (Arneodo et al., 2003), we show that the long-range correlations and anti-correlations respectively observed in the roughness fluctuations of the mammograms of dense and fatty normal breasts, strikingly vanish in the malignant tumor region. Note that most existing computer-aided diagnostic (CAD) methods (Karahaliou et al., 2008; Ayer et al., 2010; Tsai et al., 2011; Häberle et al., 2012; Meselhy Eltoukhy et al., 2012) are designed for texture analysis or feature extraction with the prerequisite that the background roughness fluctuations of normal breast mammograms are homogeneous and uncorrelated. In contrast, we propose characterizing correlations in mammogram roughness fluctuations via the estimate of a density fluctuation index *H* as an innovative and effective discrimination method that will assist in early breast cancer detection.

## 2. Methods of analysis

The wavelet transform (WT) is a mathematical microscope (Arneodo et al., 1988, 1995, 2008; Muzy et al., 1991, 1994) suitable to the analysis of complex non-stationary time-series, such as those found in genomics (Nicolay et al., 2007; Arneodo et al., 2011; Audit et al., 2013) and physiological systems (Ivanov et al., 1999, 2001; Goldberger et al., 2002; Richard et al., 2015), thanks to its ability to be blind to low-frequency trends in the analyzed signal Σ(*t*). Its generalization in two dimensions (2D) is used in image processing (Mallat, 1998; Arneodo et al., 2003, 2008; Antoine et al., 2008), including applications in cellular biology (Khalil et al., 2007; Snow et al., 2008; Goody et al., 2010; Grant et al., 2010) and medicine (Kestener et al., 2001; Khalil et al., 2009; Batchelder et al., 2014).

### 2.1. The 1D wavelet transform (time-frequency analysis)

The WT is a time-scale decomposition method which consists in expanding signals in terms of wavelets constructed from a single function, the “analyzing wavelet” ψ, by means of translations and dilations. The WT of a real-valued function Σ(*t*) is defined as (Mallat, 1998)

(1) $$W_{\psi }[\Sigma ](t_{0},a)=\frac{1}{a}\int_{-\infty }^{+\infty }\Sigma (t)\psi (\frac{t-t_{0}}{a})dt\;,$$

where *t*_0_ is a time parameter and *a* (> 0) a scale parameter (inverse of frequency). By choosing a wavelet ψ whose *n* + 1 first moments are zero [∫*t*^*m*^ψ(*t*)*dt* = 0, 0 ≤ *m* ≤ *n*], one makes the WT microscope blind to order-*n* polynomial behavior, a prerequisite for multifractal fluctuations analysis (Muzy et al., 1991, 1994; Arneodo et al., 1995, 2008).

### 2.2. The 2D wavelet transform (space-scale analysis)

With an adapted analyzing wavelet, one can reformulate Canny's multiscale edge detection in terms of a 2D wavelet transform (Mallat, 1998). The underlying strategy consists in smoothing the discrete image data by convolving it with a filter prior to computing the gradient of the smoothed image. Let us define two wavelets as the partial derivatives with respect to *x* and *y* of a 2D-smoothing function ϕ(*x*, *y*) (Mallat, 1998; Arneodo et al., 2003):

(2) $$\psi_{1}(x,y)=\frac{\partial \phi (x,y)}{\partial x}\text{ and }\psi_{2}(x,y)=\frac{\partial \phi (x,y)}{\partial y}.$$

The WT of *I*(*x*, *y*) with respect to ψ_1_ and ψ_2_ has two components and therefore can be expressed in a vectorial form:

(3) $$\begin{array}{l} W_{\psi }[I](x_{0},a)=\left\{\begin{array}{c} W_{\psi_{1}}[I]=\frac{1}{a^{2}}\int f(x)\psi_{1}(\frac{x-x_{0}}{a})d^{2}x\\ W_{\psi_{2}}[I]=\frac{1}{a^{2}}\int f(x)\psi_{2}(\frac{x-x_{0}}{a})d^{2}x \end{array}\right\},\\ \;=\nabla \{\phi_{x_{0},a}*I\}, \end{array}$$

where **x_0_** is a space parameter and *a*(> 0) a scale parameter. From Equation (3), we can compute the modulus and argument of the WT:

(4) $$M_{\psi }[I](x_{0},a)={\left[{\left(W_{\psi_{1}}[I](x_{0},a)\right)}^{2}+{\left(W_{\psi_{2}}[I](x_{0},a)\right)}^{2}\right]}^{1/2},$$

and

(5) $$A_{\psi }[I](x_{0},a)=\text{Arg}[W_{\psi_{1}}[I](x_{0},a)+iW_{\psi_{2}}[I](x_{0},a)].$$

### 2.3. The wavelet transform modulus maxima method

The wavelet transform modulus maxima (WTMM) method was originally developed to generalize box-counting techniques (Arneodo et al., 1987) and to circumvent the limitations of the structure function method to perform multifractal analysis of 1D velocity signal in fully-developed turbulence (Muzy et al., 1991, 1993, 1994; Bacry et al., 1993; Arneodo et al., 1995). It proved to be a reliable method to estimate scaling exponents and multifractal spectra (Muzy et al., 1994; Delour et al., 2001; Audit et al., 2002). The WTMM method was generalized in 2D for the multifractal analysis of rough surfaces (Arneodo et al., 2000, 2003; Decoster et al., 2000; Roux et al., 2000), and for the analysis of 3D scalar and vector fields (Kestener and Arneodo, 2003, 2004; Arneodo et al., 2008). Successful applications have already been reported in various area of fundamental research (Muzy et al., 1994; Arneodo et al., 1995, 2002, 2008, 2011). In the context of present study, the 1D WTMM method demonstrated the multifractality in physiologic dynamics and its breakdown with disease (Ivanov et al., 1999, 2001; Goldberger et al., 2002), whereas the 2D WTMM method was used to detect microcalcifications and shown to have great potential to assist in cancer diagnosis from digitized mammograms (Kestener et al., 2001; Arneodo et al., 2003; Batchelder et al., 2014).

In 1D, the WTMM method consists in computing the WT skeleton defined, at each fixed scale *a*, by the local maxima L(*a*) of the WT modulus M(*t*, *a*) = |*W*_ψ_(*t*, *a*)|. These WTMM are positioned across scales on curves called maxima lines (Figure S1). In 2D, these WTMM lie, for a given scale, on connected chains called maxima chains (Figures S2A–C). Considering the points along these maxima chains where M(**x**, *a*) is locally maximum, we define the so-called WTMMM. The WTMMM are then linked through scales to form the WT skeleton (Figure S2D). Along these maxima lines the WTMM (resp. WTMMM) behave as *a*^*h*(*t*)^ (resp. *a*^*h*(**x**)^), where *h*(*t*) (resp. *h*(**x**)) is the Hölder exponent characterizing the singularity of Σ (resp. *I*) at time *t* (resp. position **x**) (Muzy et al., 1994; Arneodo et al., 2003, 2008). The multifractal formalism amounts to quantify statistically the contributions of each Hölder exponent value via the computation of the singularity spectrum defined as the fractal dimension *D*(*h*) of the set of points *t* (resp. **x**) where *h*(*t*)(resp. *h*(**x**)) = *h*. This spectrum can be derived from the scaling behavior of partition functions defined in terms of WT coefficients (Muzy et al., 1991, 1994; Arneodo et al., 1995, 2008, 2003):

(6) $$Z(q,a)=\sum_{l\in L(a)}M{\left(\cdot ,a\right)}^{q}\sim a^{\tau (q)},$$

where *q* ∈ ℝ. Then, from the scaling function τ(*q*), *D*(*h*) is obtained by a Legendre transform:

(7) $$D(h)=\underset{q}{\min}[qh-\tau (q)].$$

In practice, to avoid instabilities in the estimation of the singularity spectrum *D*(*h*) through the Legendre transform, we instead compute the following expectation values (Muzy et al., 1994; Arneodo et al., 1995, 2003)

(8) $$h(q,a)=\frac{\partial }{\partial q}\ln(Z(q,a))=\sum_{l\in L(a)}\ln\left(M(\cdot ,a)\hat{W}(q,l,a)\right),$$

and

(9) $$\begin{array}{l} D(q,a)=q\frac{\partial }{\partial q}\ln(Z(q,a))-Z(q,a)\\ \;=\sum_{l\in L(a)}\hat{W}(q,l,a)\ln\left(\hat{W}(q,l,a)\right)\text{​}, \end{array}$$

where $\hat{W}\left(q,l,a\right)=\frac{M{\left(\cdot ,a\right)}^{q}}{Z\left(q,a\right)}$ corresponds to the Bolzmann weight in the analogy that connects the multifractal formalism to thermodynamics (Arneodo et al., 1995). Then, from the slopes of *h*(*q*, *a*) and *D*(*q*, *a*) vs. ln *a*, we get *h*(*q*) and *D*(*q*), and in turn the *D*(*h*) singularity spectrum as a curve parametrized by *q*.

### 2.4. Monofractal vs. multifractal functions

Homogeneous *monofractal* functions are functions with singularities of unique Hölder exponent *H*. Their τ(*q*) spectrum is a linear curve of slope *H*. Monofractal scaling indeed means that the shape of the probability distribution function (pdf) of rescaled wavelet coefficients (M(·, *a*)/*a*^*H*^) is independent of the scale *a*: $\rho_{M_{a}/a^{H}}\left(w\right)=\rho \left(w\right)$, where ρ(*w*) is a “universal” pdf (Arneodo et al., 2002, 2011). A nonlinear τ(*q*) is the signature of nonhomogeneous *multifractal* functions with Hölder exponent *h*(*t*) (resp. *h*(**x**)) fluctuating over time *t* (Muzy et al., 1991, 1994; Arneodo et al., 1995, 2008) [resp. over space **x** (Arneodo et al., 2000, 2003; Decoster et al., 2000; Roux et al., 2000)]. τ(*q*) data are generally well approximated by the log-normal quadratic spectrum:

(10) $$\tau (q)=-c_{0}+c_{1}q-c_{2}q^{2}/2,$$

where the coefficients *c*_*n*_ > 0. The corresponding singularity spectrum has a quadratic single-humped shape

(11) $$D(h)=c_{0}-{\left(h-c_{1}\right)}^{2}/2c_{2},$$

where *c*_0_ = −τ(0) is the fractal dimension of the support of singularities of Σ (resp. *I*), *c*_1_ is the value of *h* that maximizes *D*(*h*) and the *intermittency coefficient c*_2_ (Delour et al., 2001) characterizes the width of the *D*(*h*) spectrum as an indication of a change in WT coefficient statistics across scales (Muzy et al., 1991, 1994; Arneodo et al., 1995, 2002, 2003, 2008).

## 3. Description of data

### 3.1. Study design and population

Subjects were recruited for the present study from Perm Region Oncological Dispensary using procedures approved by the Local Ethics Committe (Gileva et al., 2012). Patients gave informed consent to participate in this study via recording IR thermograms and X-ray mammograms of their two mammary glands: the cancerous breast and the contralateral unaffected breast. The thermography (resp. mammography) database included 33 (resp. 30 among these 33) females, aged 37–83 (average 57 years), who all went through surgery to remove a histologically-confirmed malignant tumor (invasive ductal and/or lobular cancer) a few weeks after thermo-(mammo-)grams were recorded. The tumors were found at depths varying from 1 to 12 cm into the tissue, with a size (diameter) varying from 1.2 cm up to 6.5 cm (Table S1).

### 3.2. IR thermography imaging

Recording protocol was described in our preliminary study (Gerasimova et al., 2014). The patient's two breasts were imaged with an InSb photovoltaic (PV) detector (Joro et al., 2008). We performed imaging with the patient sitted with arms down to avoid too much discomfort. Frontal images were taken at a distance ~1 m from the patient, with an environmental room temperature ~20−22 °C. The image frame rate was 50 Hz. During the 10 min immobile imaging period, we collected 30,000 256 × 320 pixel^2^ image frames at 14 bits on a computer connected to the PV camera. We used skin surface markers as reference points for low-frequency motion correction in the analysis.

### 3.3. X-ray mammography

The mammographic procedure involved taking two X-ray images of the (two) compressed breasts of each patient, the standard medio-lateral oblique (MLO) and cranio-caudal (CC) views. Spatial resolution of the images was 50 μm per pixel. The ionizing radiation dose was 0.4 mSv per patient.

### 3.4. Data sampling

#### 3.4.1. Thermograms

**Figure 1:**
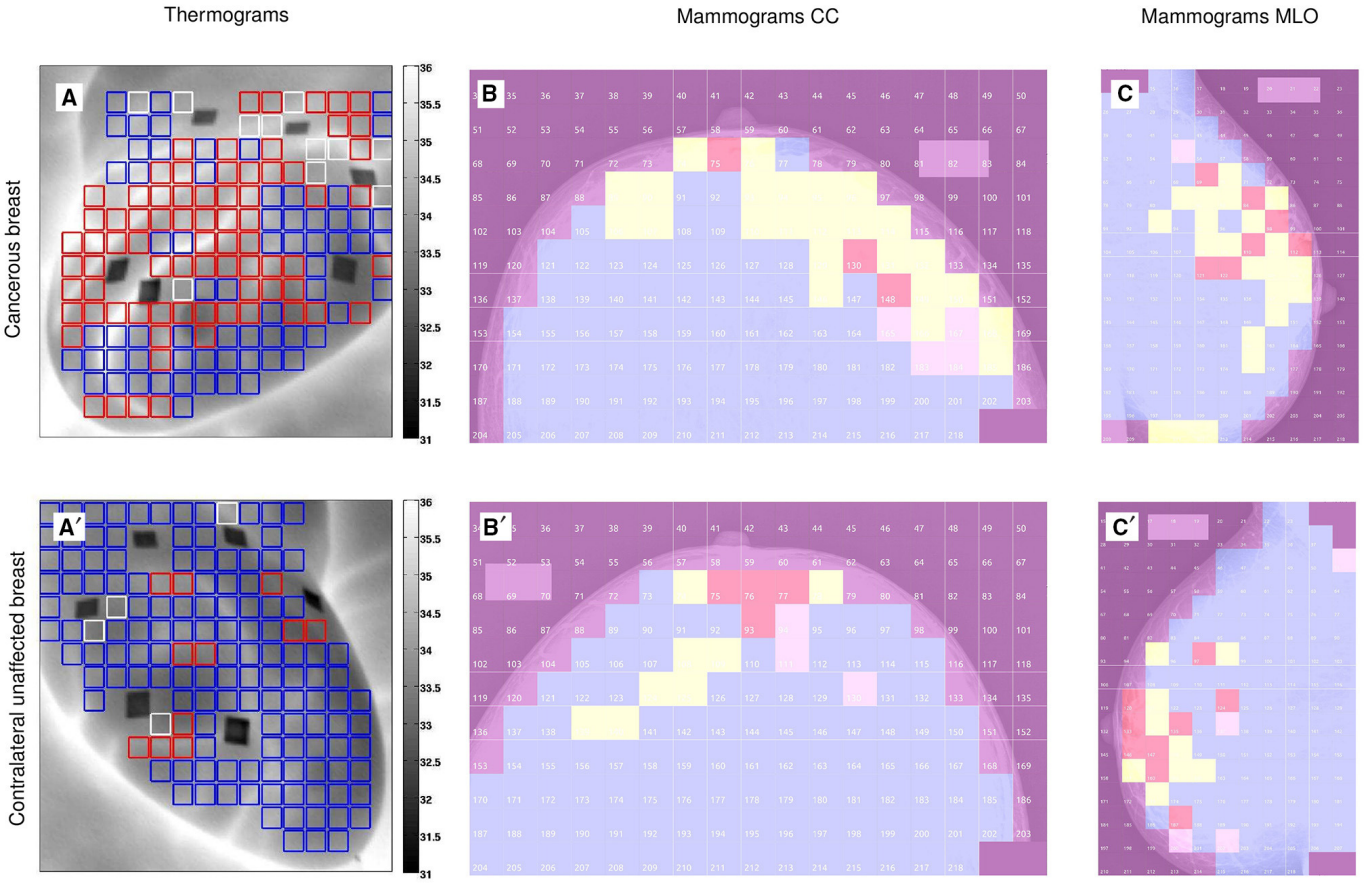
**Wavelet-based multifractal segmentation of dynamic infrared thermograms and X-ray mammograms**. Patient 20 (age 56): cancerous right breast **(A–C)** and contralateral unaffected left breast **(A′–C′)**. **(A,A′)** As estimated from the τ(*q*) spectrum of skin temperature temporal fluctuations computed with the 1D WTMM method (Figures 2D–F), 8 × 8 pixel^2^ squares spanning 10 × 10 mm^2^ were color coded according to monofractal (*c*_2_ < 0.03, red), multifractal (*c*_2_ ≥ 0.03, blue) and no scaling (white) diagnostic, where *c*_2_ is the intermittency coefficient that defines the width of the *D*(*h*) singularity spectrum (Equations 10, 11) (Gerasimova et al., 2014). **(B,B′)** As estimated from the τ(*q*) spectra of CC mammographic view computed with the 2D WTMM method (Figures 2A–C), 256 × 256 pixel^2^ squares spanning 12.8 × 12.8 mm^2^ were color coded according to monofractal *H* < 0.45 (blue), 0.45 ≤ *H* ≤ 0.55 (yellow), *H* > 0.55 (red) and no scaling (pink). **(C,C′)** Same as **(B,B′)** for MLO mammographic view.

As commonly done for noise signals (Muzy et al., 1994; Audit et al., 2002), and previously performed to analyze rainfall time-series (Venugopal et al., 2006), the 1D WTMM method was applied to the cumulative (or integral) Σ of the temperature time-series (Figure S1), using the second-order compactly supported version $\psi_{\left(3\right)}^{\left(2\right)}$ of the Mexican hat (Roux et al., 1999) (see Figure S1 in (Gerasimova et al., 2014)). The singularities with possible negative Hölder exponent −1 < *h* < 0, became singularities with 0 < *h*_*c*_ = *h* + 1 < 1 in the cumulative. We grouped single-pixel temperature time-series (Figures S1A,B) into 8 × 8 pixel^2^ squares spanning 10 × 10 mm^2^ and covering the entire breast (see Figures 1A,A′). The results thus correspond to averaged partition functions and multifractal τ(*q*) (Equation 10) and *D*(*h*) (Equation 11) spectra computed from the WT skeleton (e.g., Figures S1E,F) of 64 cumulative temperature time-series in these 8 × 8 subareas. For each breast analyzed, we have color-coded these squares according to the diagnosis obtained with the 1D WTMM method (Table 2 in Gerasimova et al., 2014): monofractal (*c*_2_ < 0.03, red), multifractal (*c*_2_ ≥ 0.03, blue) and no scaling (white) (Figures 1A,A′).

#### 3.4.2. Mammograms

For 2D WTMM analysis of mammograms, we used the isotropic Gaussian function

(12) $$\Phi (x,y)=\exp^{-(x^{2}+y^{2})/2}=\exp^{-|x{|}^{2}/2},$$

as a smoothing function, meaning that we worked with first-order (one vanishing moment) analyzing wavelets ψ_1_ and ψ_2_ (Equation 2). We divided the entire mammographic images into 360 × 360 pixels^2^ overlapping (to control edge effects) squares spanning 18 × 18 mm^2^. Subimages of the breast in each of these squares were analyzed using the 2D WTMM methodology (Arneodo et al., 2000, 2003; Decoster et al., 2000; Roux et al., 2000). For a given square, when computing the partition functions and multifractal spectra from the WT skeleton, only the central (256 × 256 pixels^2^) part that does not overlap with the central part of the neighboring squares was taken into account (Figure S2). For each breast analyzed, we have color-coded these squares according to the monofractal diagnosis obtained with the 2D WTMM method: long-range correlations (*H* > 0.55, red), anti-correlations (*H* < 0.45, blue), no-correlations (0.45 ≤ *H* ≤ 0.55, yellow), and no scaling (pink) (Figures 1B,B′,C,C′).

### 3.5. Statistical tests

Statistical analyses were performed using the R statistical package (https://cran.r-project.org/). A nonparametric Wilcoxon rank-sum test was used to calculate *p*-values when comparing CC and MLO mammograms, cancer breasts and contralateral unaffected breasts, and thermograms and mammograms. Linear correlation of data sets were analyzed via a Pearson test.

### 3.6. Software and documentation

The numerical procedure to perform the WTMM analysis of 1D signals is avaliable at http://perso.ens-lyon.fr/benjamin.audit/LastWave

LastWave is an open source software written in C. We recommend interested users read the LastWave C-Application Programming Interface documentation and to contact the corresponding author to be directed to the part of the code of most relevance to them. For 2D WTMM analysis, we adapted home-made Xsmurf software written in C, for specific application to mammograms.

## 4. Results

### 4.1. Mammographic tissue classification using the 2D WTMM method

**Figure 2:**
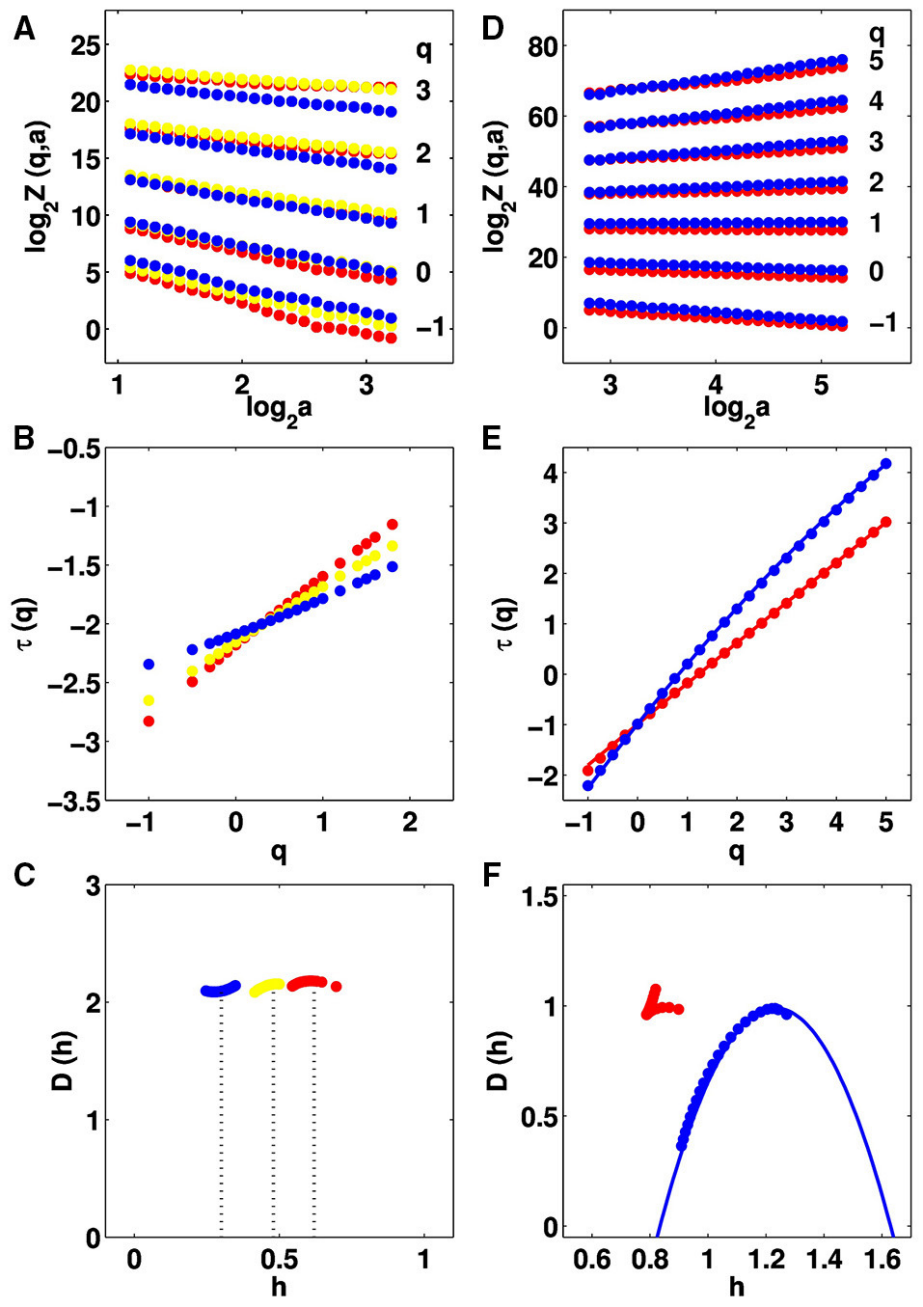
**Multifractal analysis of IR temperature time series and X-ray mammograms of patient 20**. Comparative analysis of spatial roughness fluctuations in individual 256 × 256 pixel^2^ squares in the cancerous right breast with respectively monofractal *H* < 0.45 (blue), 0.45 ≤ *H* ≤ 0.55 (yellow) and *H* > 0.55 (red): **(A)** log_2_ *Z*(*q*, *a*) vs. log_2_ *a*; **(B)** τ(*q*) vs. *q* estimated by linear regression fit of log_2_ *Z*(*q*, *a*) vs. log_2_ *a* (Equation 6) over a range of space-scales [0.7; 2.8] mm; **(C)** *D*(*h*) vs. *h*. Comparative analysis of temperature fluctuations in a 8 × 8 pixel^2^ square in the cancerous right breast (monofractal *c*_2_ < 0.03, red) and contralateral unaffected left breast (multifractal *c*_2_ ≥ 0.03, blue): **(D–F)** as **(A–C)**.

We analyzed individual 360 × 360 pixel^2^ mammographic subimages of patient breasts with malignant tumor using the 2D WTMM method (Materials and Methods). From the central 256 × 256 pixels^2^ part of the WT skeleton we computed the partition functions *Z*(*q*, *a*) (Equation 6) that were found to display nice scaling properties over a range of scales 1 ≲ log_2_ *a* ≲ 3 corresponding to [0.7 mm, 2.8 mm] for linear regression fit estimates in a logarithmic representation (Figure 2A). The scaling deteriorates when considering larger scales due to finite size effects. In the range −1 ≲ *q* ≲ 3, statistical convergence is achieved; the so-obtained τ(*q*)-spectrum is remarkably linear, as the signature of monofractal roughness fluctuations and this wherever the location of the mammographic subimages over the cancerous breast (Figure 2B). Indeed the data are well-fitted by the theoretical spectrum τ(*q*) = *qH* − 2 of monofractal rough surfaces, which are almost everywhere singular with a unique hölder exponent *h* = *H* (Arneodo et al., 2000, 2003, 2008; Decoster et al., 2000). This is confirmed when computing the *D*(*h*) singularity spectrum from the slopes *h*(*q*) and *D*(*q*) of the partition functions *h*(*q*, *a*) (Equation 8) and *D*(*q*, *a*) (Equation 9) vs. ln *a* (Figure S3). *h*(*q*) and *D*(*q*) do not significantly depend on *q*, meaning that the *D*(*h*) singularity spectrum reduces to a single point *D*(*h* = *H*) = 2 (Figure 2C). Actually, what possibly changes when investigating different regions of cancerous breasts is the Hurst exponent *H* determined by the WTMM method (Figures 2B,C). As previously noted in a preliminary study (Kestener et al., 2001; Arneodo et al., 2003), we recovered *H* ≃ 1/3 in regions of fatty breast tissues as an indication of antipersistent (anti-correlated) roughness fluctuations, while dense tissues more likely displayed monofractal scaling with *H* ≃ 2/3 as an indication of persistent long-range correlations. But what was undetermined in previous analysis, was the existence of breast regions where the estimated Hurst exponent *H* ≃ 1/2, the hallmark of uncorrelated monofractal rough surfaces (Figures 2B,C). Importantly, the loss of correlations in roughness fluctuations is mainly observed in the region of the breast where the malignant tumor is located.

### 4.2. Segmentation of breast mammograms into physiologically altered (risky) and normal regions: comparative analysis of the CC and MLO mammographic views

**Figure 3:**
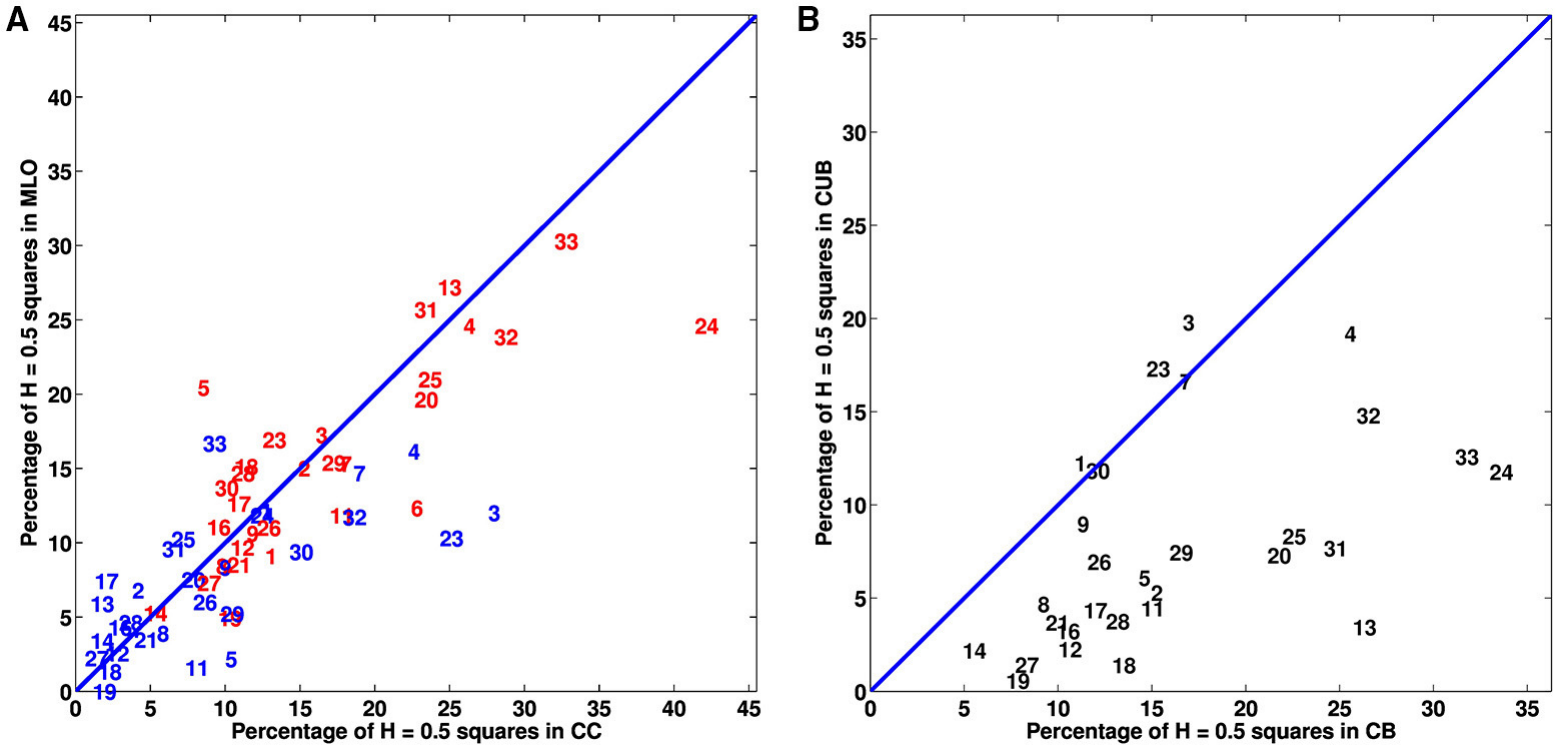
**Analysis of the percentage of monofractal uncorrelated *H* = 0.5 256 × 256 pixel^2^ squares in the mammograms of the breasts of 30 patients with breast cancer. (A)** Percentage of *H* = 0.5 squares in cancerous (red) and contralateral unaffected (blue) breasts: MLO view vs. CC view. **(B)** Mean percentage of *H* = 0.5 squares in MLO and CC views: contralateral unaffected breast (CUB) vs. cancerous breast (CB). In **(A,B)**, the numbers correspond to patient numbers defined in Table S1.

**Figure 4:**
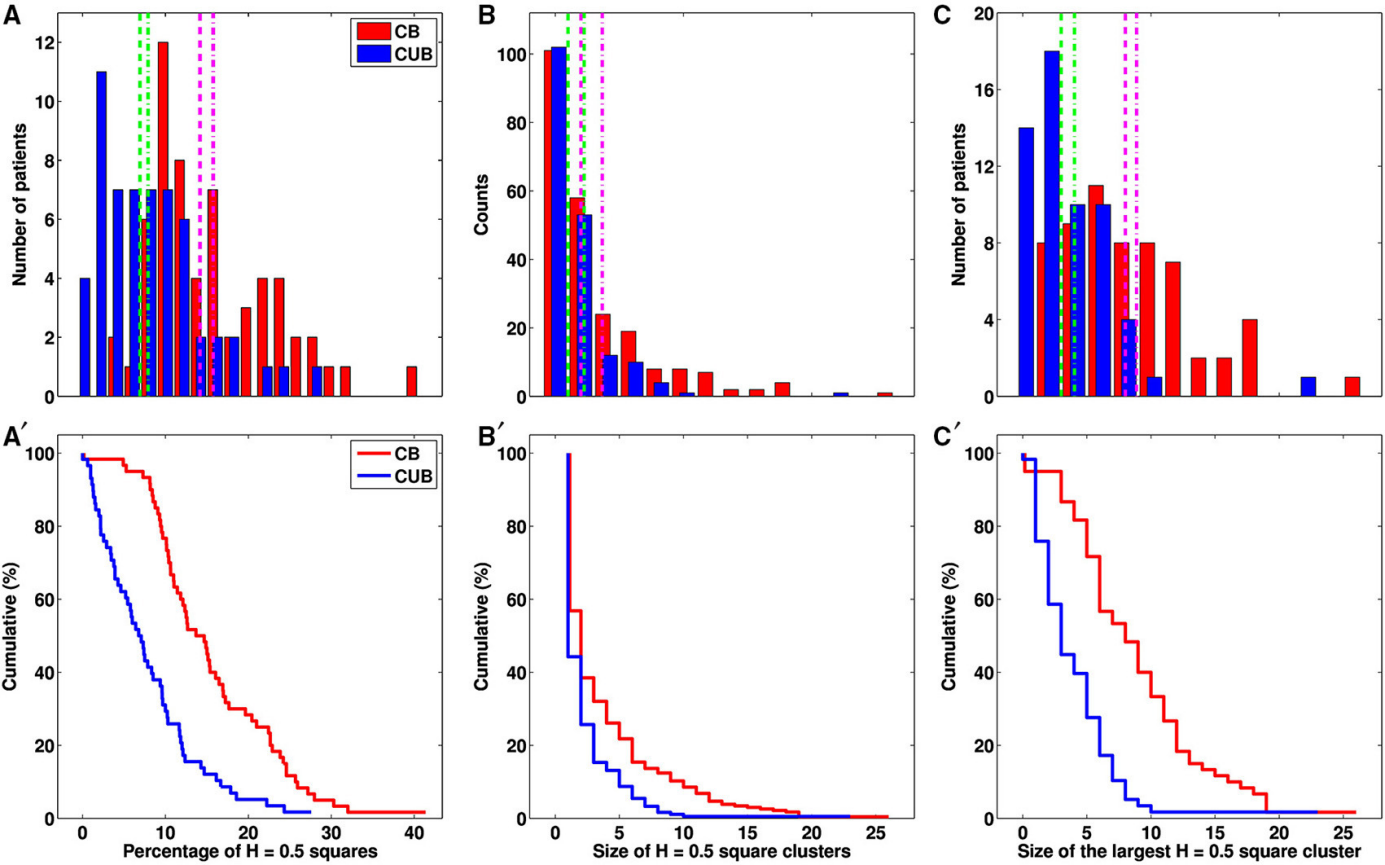
**Differential monofractal *H* = 0.5 signature on the (CC and MLO) mammograms of cancerous breasts (CB) and contralateral unaffected breasts (CUB). (A)** Histograms of the percentage of squares in the mammograms of the CBs (red) and CUBs (blue) of 30 patients with breast cancer. **(B)** Histograms of the size of *H* = 0.5 square clusters (see text) in CBs (red) and CUBs (blue). **(C)** Histograms of the size of the largest *H* = 0.5 square cluster in CBs (red) and CUBs (blue). **(A′–C′)** same as **(A–C)** for the corresponding cumulative distribution functions. Clusters are defined by squares sharing a common edge or corner. In **(A–C)**, the pink and green vertical lines correspond to the mean (dashed line) and median (dashed-dotted line) of the histogram obtained for CBs and CUBs respectively.

We extended our wavelet-based multifractal analysis of CC and MLO mammographic images to the entire sets of 256 × 256 pixels^2^ squares that cover every breast of the 30 (among 33) patients that proceeded through X-ray mammography prior to surgery. As shown in Figures 1B,B′,C,C′ for patient 20, we color-coded each of these squares according to the parameter *H* estimated with the 2D WTMM method: (i) *H* < 0.45, blue (“fatty”) anti-correlated rough surface, (ii) *H* > 0.55, red (“dense”) long-range correlated rough surface, (iii) 0.45 ≤ *H* ≤ 0.55, yellow uncorrelated rough surface, and pink when no convincing scaling was observed in the considered square. For the two mammographic views of each breast, we calculated the numbers of blue (*N*_*b*_), red (*N*_*r*_), yellow (*N*_*y*_) and pink (*N*_*n*_) squares and corresponding percentages of breast coverage (Table S2). We first confirmed that, except in a minority of squares, monofractal scaling was clear. For the cancerous breasts, the mean percentage of no-scaling pink squares was 6.5% (resp. 5.7%) in CC (resp. MLO) mammograms, which was consistent with the mean percentage 5.9% (resp. 6.1%) in CC (resp. MLO) mammograms obtained for the (a priori healthy) contralateral unaffected breasts. Most of the patients had breasts consisting of mostly fatty tissues, with a majority of anti-correlated blue squares covering on average 70.9% (resp. 72.4%) of CC (resp. MLO) mammograms of the cancerous breasts and 79.1% (resp. 80.1%) in CC (resp. MLO) mammograms of the contralateral unaffected breasts. This excess was compensated by a rather small percentage of long-range correlated red squares 6.4% (resp. 6.5%) in CC (resp. MLO) mammograms of the cancerous breasts, as well as in the CC (resp. MLO) mammograms of the contralateral unaffected breasts [6.5% (resp. 6.5%)]. A similar agreement was observed between the percentages of uncorrelated yellow squares (further called *H* = 0.5 squares) in the CC and corresponding MLO mammograms of each breast of each patient (Figure 3A and Table S2). However, the number (Figures S4, S5A,A′) and percentage (Figures 3A, 4A,A′) of uncorrelated *H* = 0.5 squares in the cancerous breast mammograms were both larger than in the contralateral unaffected breast mammograms. In the CC (resp. MLO) view, *N*_*y*_/*N*_*Tot*_ ranges in the interval [4.5%, 41.4%] (resp. [4.9%, 32%]) with a mean percentage 16.1% (resp. 15.4%) for the breasts with the malignant tumor, as compared to the interval [0.6%, 27%] (resp. [0%, 16.7%]) and mean percentage 8.5% (resp. 7.3%) for the opposite breasts. A statistical comparison between cancerous breasts and contralateral unaffected breasts was performed using a Wilcoxon rank-sum test which yielded *p* < 10^−4^ and 10^−5^ for the moduli of the values in Figure 3A and Figure S4A, respectively. Moreover, the moduli of values for both cancerous breasts and contralateral unaffected breasts in CC and MLO mammograms exhibited linear correlation. In Figures 4A,A′, cancerous breasts and contralateral unaffected breasts were different with *p* < 10^−8^. This statistically significant excess of *H* = 0.5 squares in the mammograms of cancerous breasts is evidence that the loss of correlations in the mammographic spatial density fluctuations can be used as an indicator of malignancy.

### 4.3. Comparative statistical analysis of mammogram roughness fluctuations in cancerous and contralateral unaffected breasts

**Figure 5:**
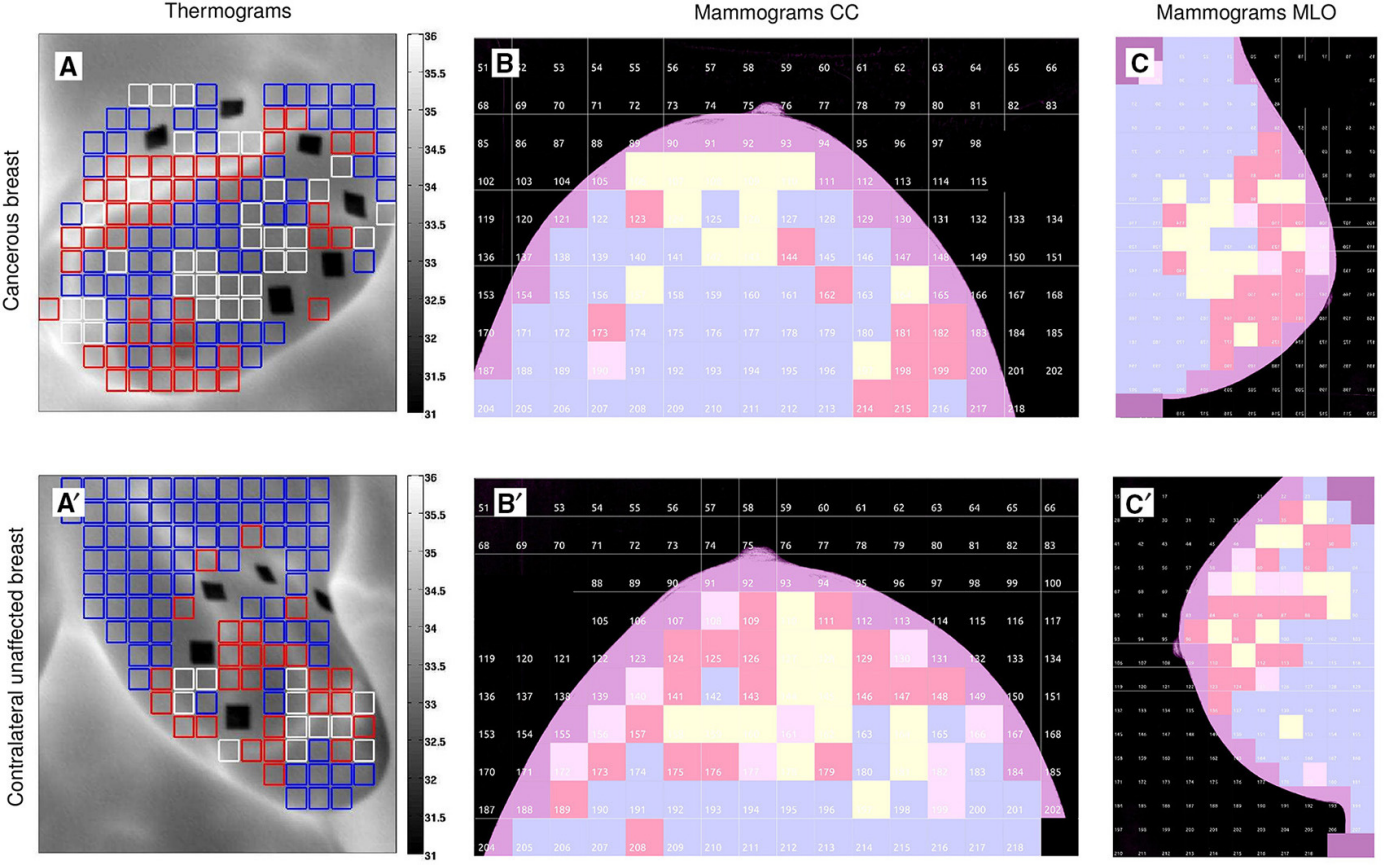
**Wavelet-based multifractal segmentation of dynamic infrared thermograms and X-ray mammograms**. Same as Figure 1 but for patient 7 (age 47): cancerous right breast **(A–C)** and contralateral unaffected left breast **(A′–C′)**.

Asymmetry of patient's breast can be a sign of breast cancer. When comparing the percentages of uncorrelated *H* = 0.5 squares on both the cancerous and contralateral unaffected breast mammograms of each patient, we found that a majority (25/30) have more *H* = 0.5 squares (average over CC and MLO views) on the cancerous breast (Figure 3B and Figure S4B and Table S2), including patients 20 (Figure 1), 17 (Figure S6), 24 (Figure S7), and 29 (Figure S8). For the 5 patients left including patients 3 (Figure S9), 7 (Figure 5) and 30 (Figure S10), an important and slightly larger (or equal) percentage of *H* = 0.5 squares was found in the second breast as a probable indication of some physiological and architectural changes in the undiagnosed opposite breast. When looking at the excess of *H* = 0.5 squares in the cancerous breasts we realized that these uncorrelated squares were not sparsely distributed all over the breast, but were concentrated in specific regions likely surrounding the underlying malignant tumor (Figures 1B,C and Figures S6B,C, S7B,C, S8B,C). To quantify this inhomogeneous distribution, we investigated the size distribution of *H* = 0.5 clusters defined as *H* = 0.5 squares sharing a common edge or corner (Table 1). If cancerous and contralateral unaffected breasts display about the same number of isolated *H* = 0.5 squares (singlet), the former are comprised of clusters with sizes larger than 2 and up to 26 (patient 13). The corresponding histogram of *H* = 0.5 cluster size displays a long tail in cancerous breasts that has no counterpart in the contralateral unaffected breasts (Figure 4B). As seen on the cumulative histogram (Figure 4B′), more than 20% of *H* = 0.5 clusters have a size larger than 5 in the cancerous breast as compared to only 8.7% in the contralateral unaffected breasts. This clustering effect is even more pronounced on the histogram of the size of the largest *H* = 0.5 square cluster (Figure 4C), with 33.3% of the cancerous breasts with a largest *H* = 0.5 square cluster bigger than 10 as compared to only 3.4% for the contralateral unaffected breasts (Figure 4C′). Using Wilcoxon rank-sum test to compare cancerous breasts and contralateral unaffected breasts in Figures 4A–C yielded *p* < 10^−8^, 10^−3^, and 10^−8^, respectively. Note that the only contralateral unaffected breast with a *H* = 0.5 square cluster of size 22 is the second breast of patient 3 that was previously shown to have more *H* = 0.5 squares that the breast with the malignant tumor (Figure S9). Quite similar statistical results were obtained when defining *H* = 0.5 clusters from squares sharing a common edge only (Figures S5B,B′,C,C′ and Table S3).

**Table 1:** **Statistics of monofractal uncorrelated *H* = 0.5 yellow square clusters in the CC and MLO mammographic views of the cancerous and contralateral unaffected breasts of our patients with breast cancer**.

	**Cancerous breast**			**Contralateral unaffected breast**		
	**Total N_*y*_**	**N_*cluster*_**	**Size_*cluster*_**	**Total N_*y*_**	**N_*cluster*_**	**Size_*cluster*_**
1-CC	10	3	5 4 1	11	4	7 2 1 1
1-MLO	8	6	3 1 1 1 1 1	12	3	8 2 2
2-CC	7	2	6 1	2	2	1 1
2-MLO	9	1	9	4	2	2 2
3-CC	13	3	11 1 1	24	2	23 1
3-MLO	15	4	9 2 2 2	12	9	2 2 2 1 1 1 1 1 1
4-CC	14	5	5 4 2 2 1	12	2	9 3
4-MLO	15	3	12 2 1	10	5	5 2 1 1 1
5-CC	8	5	3 2 1 1 1	10	5	4 2 2 1 1
5-MLO	19	4	11 6 1 1	2	2	1 1
6-CC	24	5	18 3 1 1 1	0	0	0
6-MLO	19	3	17 1 1	0	0	0
7-CC	12	4	9 1 1 1	13	2	10 3
7-MLO	15	4	11 2 1 1	11	5	3 3 3 1 1
8-CC	19	7	6 4 3 2 2 1 1	11	3	8 2 1
8-MLO	17	3	14 2 1	8	6	2 2 1 1 1 1
9-CC	12	7	5 2 1 1 1 1 1	10	3	6 3 1
9-MLO	12	2	10 2	9	6	3 2 1 1 1 1
11-CC	9	3	6 2 1	3	2	2 1
11-MLO	9	6	3 2 1 1 1 1	1	1	1
12-CC	15	4	12 1 1 1	3	3	1 1 1
12-MLO	15	6	6 4 2 1 1 1	4	4	1 1 1 1
13-CC	23	3	19 2 2	1	1	1
13-MLO	28	3	26 1 1	7	4	3 2 1 1
14-CC	4	2	3 1	1	1	1
14-MLO	6	3	2 2 2	4	1	4
16-CC	10	4	6 2 1 1	3	1	3
16-MLO	17	3	15 1 1	6	2	4 2
17-CC	9	4	6 1 1 1	1	1	1
17-MLO	14	2	13 1	6	2	5 1
18-CC	14	8	5 3 1 1 1 1 1 1	2	1	2
18-MLO	18	5	8 6 2 1 1	2	2	1 1
19-CC	10	1	10	1	1	1
19-MLO	6	1	6	0	0	0
20-CC	24	2	19 5	8	3	6 1 1
20-MLO	22	2	19 3	9	3	7 1 1
21-CC	14	5	9 2 1 1 1	5	4	2 1 1 1
21-MLO	12	7	2 2 2 2 2 1 1	5	5	1 1 1 1 1
23-CC	5	3	3 1 1	9	2	8 1
23-MLO	11	4	4 4 2 1	7	2	6 1
24-CC	24	3	13 6 5	7	4	3 2 1 1
24-MLO	15	3	12 2 1	9	3	7 1 1
25-CC	19	5	12 4 1 1 1	5	4	2 1 1 1
25-MLO	17	4	6 5 5 1	9	3	5 3 1
26-CC	4	1	4	7	4	3 2 1 1
26-MLO	10	1	10	6	1	6
27-CC	13	4	10 1 1 1	1	1	1
27-MLO	11	6	4 3 1 1 1 1	4	3	2 1 1
28-CC	13	6	5 2 2 2 1 1	4	3	2 1 1
28-MLO	16	6	8 4 1 1 1 1	6	2	5 1
29-CC	14	3	7 6 1	9	4	5 2 1 1
29-MLO	14	3	6 6 2	5	1	5
30-CC	8	4	2 2 2 2	12	6	3 3 2 2 1 1
30-MLO	13	3	11 1 1	9	3	6 2 1
31-CC	12	2	9 3	3	2	2 1
31-MLO	18	4	8 7 2 1	7	3	3 3 1
32-CC	40	8	16 7 4 4 4 2 2 1	30	13	6 5 3 3 3 2 2 1 1 1 1 1 1
32-MLO	36	11	12 6 4 3 3 3 1 1 1 1 1	19	10	5 4 2 2 1 1 1 1 1 1
33-CC	8	3	5 2 1	4	4	1 1 1 1
33-MLO	10	2	7 3	8	2	7 1
Average	14.3	3.9	8.9	7.1	3.2	4.0
St. dev.	6.9	2.0	5.1	5.5	2.3	3.5

*N_y_ = number of H = 0.5 squares; N_cluster_ = number of H = 0.5 square clusters; Size_cluster_ = number of H = 0.5 squares in each cluster. Clusters are defined by squares sharing a common edge or corner*.

### 4.4. Comparative multifractal analysis of the skin temperature dynamics and mammogram spatial roughness fluctuations

For the same cohort of patients, the analysis of dynamic IR thermograms put into light some significant change in the nature of temporal fluctuations of breast skin temperature in cancerous breasts (Gerasimova et al., 2013, 2014). When using the 1D WTMM method, the computation of the partition functions *Z*(*q*, *a*) (Equation 6), *h*(*q*, *a*) (Equation 8) and *D*(*q*, *a*) (Equation 9) revealed that, besides the cardiogenic and vasomotor perfusion oscillations, the temperature dynamics display some scale invariant background component that extends from the characteristic human respiratory frequency (≳ 0.3 Hz) up to the cross-over frequency (≲ 4 Hz) toward instrumental white noise (Figure 2D). For normal breasts as well as in healthy regions of cancerous breasts, the τ(*q*) spectrum is definitely non-linear (Figure 2E) and accordingly the *D*(*h*) spectrum has a single humped shape (Figure 2F), the hallmark of multifractal scaling. By contrast, in the region of the malignant tumor in cancerous breasts the τ(*q*) spectrum is nearly linear and the *D*(*h*) spectrum reduces to a single point as the signature of monofractal scaling. As illustrated in Figures 1A,A′ for patient 20, we have segmented the two breasts of each patient into 8 × 8 pixels squares that were further color-coded according to the monofractal (red), multifractal (blue) or no-scaling (white) diagnosis obtained with the 1D WTMM method. In good agreement with our previous segmentation of CC (Figures 1B,B′) and MLO (Figures 1C,C′) mammograms, more “risky” monofractal squares (49.7% coverage) were found in the cancerous breast (Figure 1A) than in the contralateral unaffected breast (7.7%) (Figure 1A′). In addition to their number, the location and clustering of these “risky” squares, where the multifractal complexity of temperature fluctuations is lost, correlate with the clustering of the “risky” *H* = 0.5 squares in CC and MLO mammograms indicating some additional evidence of loss of correlations in spatial density fluctuations in the upper outer quadrant of the right breast of patient 20 where the underlying tumor is located.

**Figure 6:**
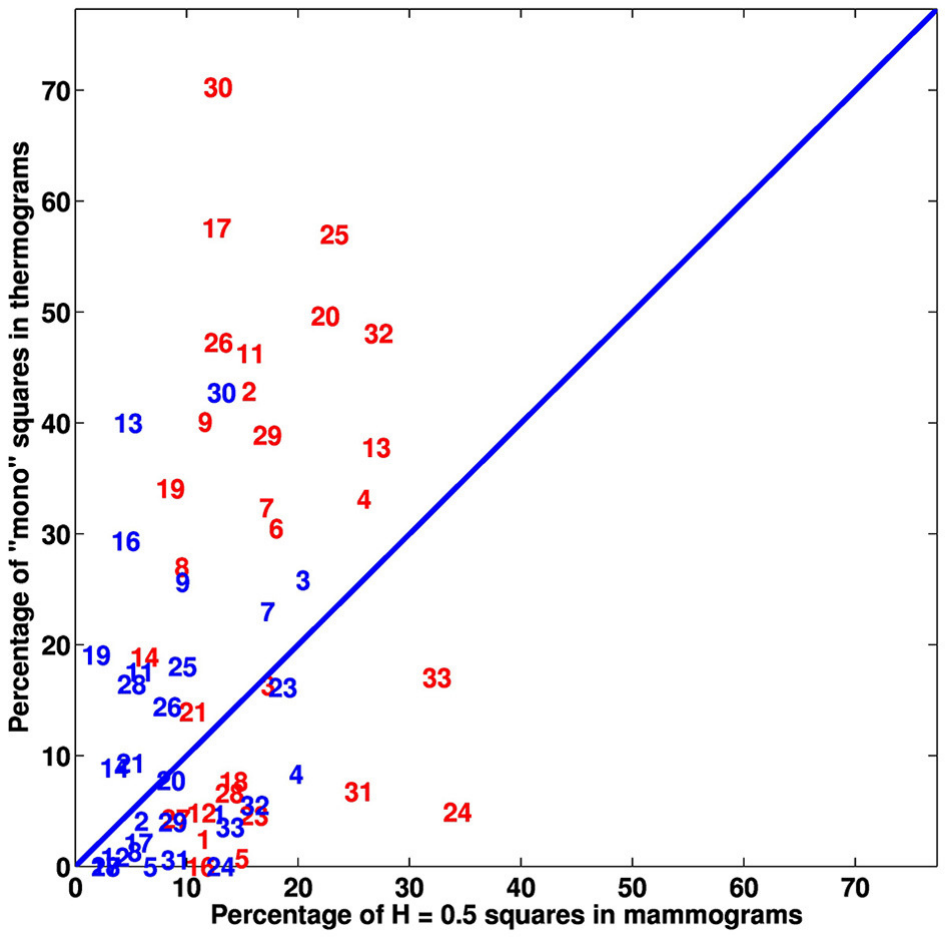
**Comparative analysis of thermograms and (CC and MLO averaged) mammograms**. Percentage of monofractal squares in thermograms vs. percentage of *H* = 0.5 squares in mammograms of the cancerous (red) and contralateral unaffected (blue) breasts of the 30 patients with breast cancer. The numbers correspond to patient numbers defined in Table S1.

**Figure 7:**
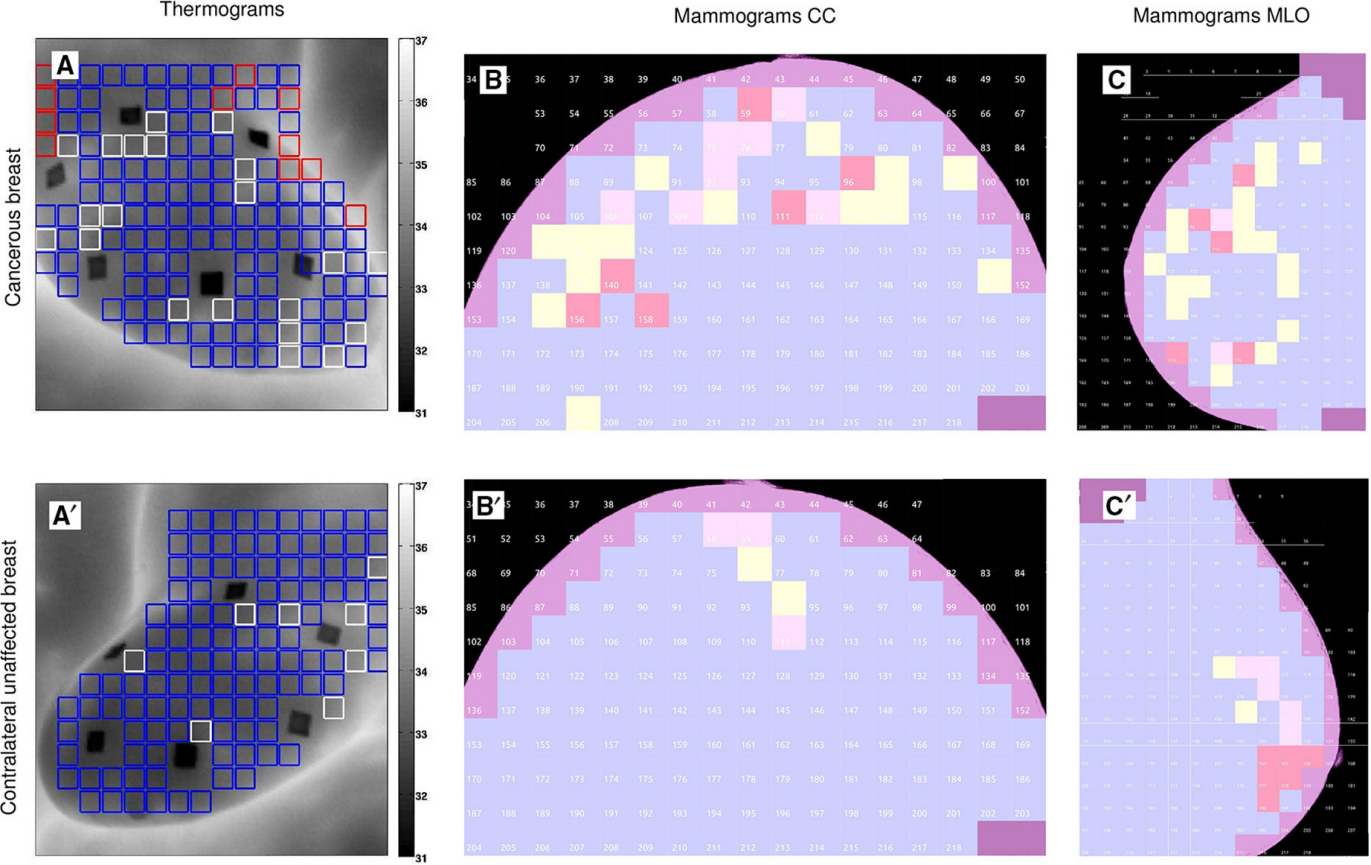
**Wavelet-based multifractal segmentation of dynamic infrared thermograms and X-ray mammograms**. Same as Figure 1 but for patient 18 (age 37): cancerous left breast **(A–C)** and contralateral unaffected right breast **(A′–C′)**.

The results obtained for patient 20 are quite representative of the outcome of our overall comparative analysis of IR thermograms and X-ray mammograms for our set of 30 patients (Figure 6, Figure S11). The use of Wilcoxon rank-sum test to compare cancerous breasts and contralateral unaffected breasts in Figure 6 and Figure S11 yielded *p* < 10^−4^ and 10^−3^, respectively. But both types of breasts did not exhibit linear correlation between mammogram and thermogram data. Most patients have consistent large numbers (breast coverage ≳ 10%) of “risky” uncorrelated *H* = 0.5 (yellow) squares in mammograms and monofractal (red) squares in thermograms of their cancerous breast as compared to their contralateral unaffected breast (breast coverage ≲ 10%), including patients 17 (Figure S6) and 29 (Figure S8). Note that for the subset of (5) patients, including patients 3 (Figure S9), 7 (Figure 5), and 30 (Figure S10), who were shown to have also a lot of “risky” *H* = 0.5 squares on their contralateral unaffected breast mammograms, they also have a lot of risky monofractal squares on the thermograms of their two breasts which might be an additional sign of the possible extension of cancer to their second breast. Among the patients with few “risky” monofractal squares (≲ 10%) on their IR thermograms of their cancerous breast, 4 correspond to rather deep tumors, namely patients 12 (size 1.8 cm, depth 12 cm), 16 (3.4 cm, 7 cm), 18 (3.49 cm, 6 cm) (Figure 7) and 28 (3.49 cm, 8 cm). For these 4 patients the percentage of “risky” *H* = 0.5 squares on their corresponding cancerous breast mammograms is significantly high (≳ 10%), meaning that, although imperceptible in temperature dynamics at the skin surface, the change in the microenvironment of these deep tumors turns out to be detectable with X-ray mammography. Let us also mention that if the Paget disease of the nipple of the left breast of patient 24 (Figure S7) was hardly identified in the IR thermograms with only few (breast coverage 4.9%), but well localized, “risky” monofractal squares (Figure S7A), it could not be missed on the CC (Figure S7B) and MLO (Figure S7C) mammograms, with respective 41.4% and 24% coverages by “risky” uncorrelated *H* = 0.5 squares.

### 4.5. Comparative analysis of the mammograms of a cancerous breast before and 11 months after surgery: a control experiment

**Figure 8:**
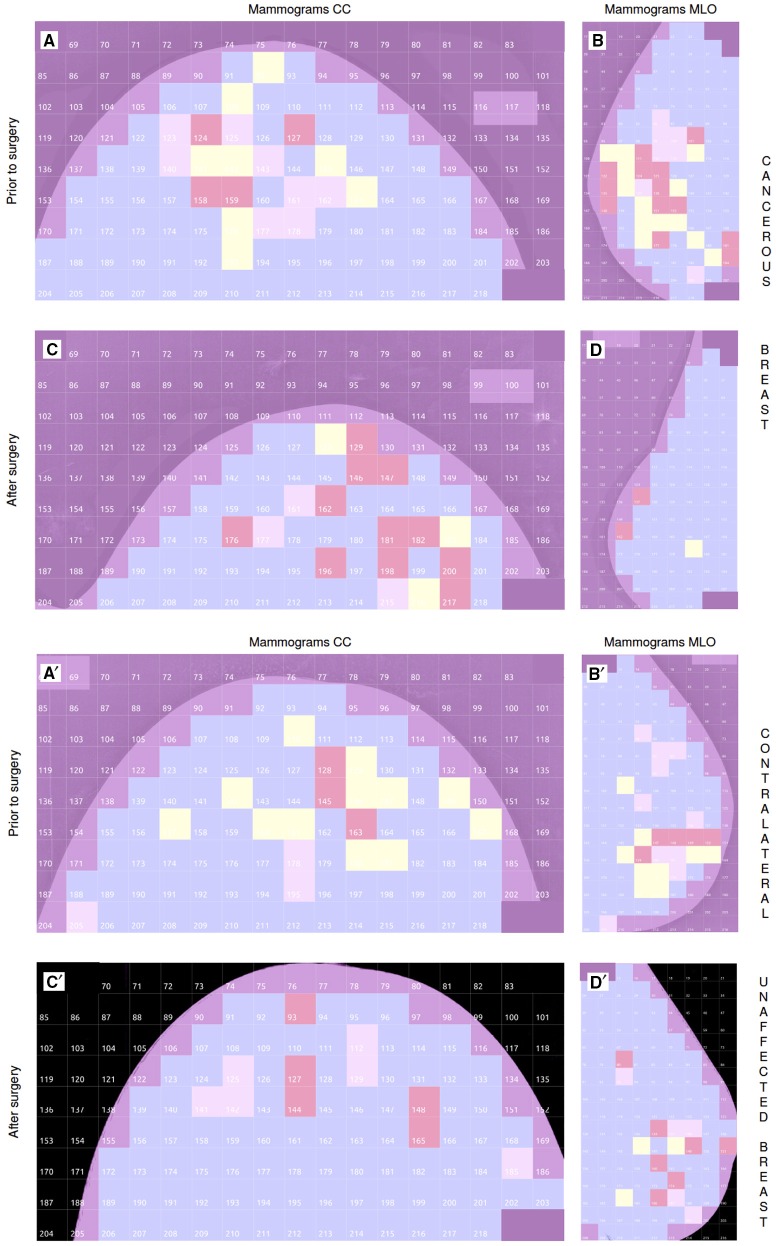
**Comparative multifractal analysis of the mammograms of the cancerous breast of patient 30 (age 54) before surgery (A,B) and 11 months after surgery (C,D)**. The color coding of the 256 × 256 pixel^2^ squares in the CC **(A,C)** and MLO **(B,D)** mammogram views is the same as in Figures 1B,B′,C,C′. **(A′–D′)** Similar comparative multifractal analysis of the mammograms of the contralateral unaffected breast of patient 30.

As a control, we reproduced our 2D WTMM multifractal analysis on the mammograms of the two breasts of patient 30 that were recorded 11 months after surgical treatment of invasive ductal carcinoma of the right breast. For both mammographic views, the number of “risky” *H* = 0.5 squares in the cancerous breast was reduced from *N*_*y*_ = 8 (Figure 8A) to 3 (Figure 8C) in CC mammograms, and from *N*_*y*_ = 13 (Figure 8B) to 1 (Figure 8D) in MLO mammograms. However, less expected was the quite similar quantitative reduction observed in the other breast of patient 30, who was one of the 5 patients having slightly more *H* = 0.5 squares on the contralateral unaffected breast than on the cancerous breast (Figure S10): *N*_*y*_ = 12 (Figure 8A′) to 0 (Figure 8C′) in CC mammograms, and *N*_*y*_ = 9 (Figure 8B′) to 3 (Figure 8D′) in MLO mammograms. These observations suggest that the surgery and associated therapeutic treatment were efficient with regards to removing the malignant tumor, as well as of its tumorigenic microenvironment including cancer stem cells. They also attest to some regeneration of the breast tissue, probably via the activation of quiescent epithelial stem cells housed in the ducts, and which were shown to exhibit a high proliferative, self-renewal and morphogenic capacity in culture (Villadsen et al., 2007). The underlying recovery mechanisms appear to be engaged and operating not only in the cancerous breast but also in the other breast which contained an above average number of “risky” *H* = 0.5 squares on both mammographic views.

## 5. Conclusions

To summarize, we showed that the wavelet-based multifractal analysis of X-ray mammograms was able to detect the presence of an internal malignant tumor via some loss of correlations in the breast density spatial fluctuations. We demonstrated that this important change in mammogram roughness fluctuations strongly correlated with some drastic simplification of temperature dynamics recorded with an IR camera, from multifractal to homogeneous monofractal temporal fluctuations. The nature of this study was exploratory, with a data set limited to females who all went through surgery to remove the histologically confirmed malignant tumor of rather large size (diameter between 1.2 and 6.5 cm). To our knowledge, our study is the first to report on the observation of some physiological alteration and architectural disorganization of the microenvironmet of breast tumors using classical screening techniques, including the currently, most used X-ray mammography. Since the alteration of the niche surrounding a breast tumor is likely to correspond to a transition from an antitumorigenic to a tumorigenic microenvironment that probably preceeds and further facilitates the process of oncogenic transformation and tumor progression (Bissell et al., 2005; Rønnov-Jessen and Bissell, 2009; Bissell and Hines, 2011; Maguer-Satta, 2011; Lu et al., 2012), the results reported are not only novel, but show great promise toward improving early breast cancer diagnosis that is known to be critical for the treatment and survival of the patient. Of course these results deserve to be confirmed over a larger set of patients at different stages of cancer development. We just started the analysis of longitudinal studies that should bring statistical estimates of the sensitivity and specificity of our mathematical and computational approach mainly based on the estimate of the mammographic density fluctuation index *H*. This preliminary study does suggest that combining sparse and sometimes painful and quite uncomfortable mammography examinations with more frequent inexpensive, quick and painless IR thermography examinations could become an efficient routine breast cancer screening method to identify, as early as possible, women with high risk of breast cancer.

## Author contributions

Conception and design: AA, ON, AK, FA, OG. Development and methodology: AA, ON, EG, BA, AK. Acquisition of data (provided animals, acquired and managed patients, provided facilities, etc.): EG. Analysis and interpretation of data: AA, EG, BT, ZM, BA, SR, AK, FA, OG, ON. Writing, review, and/or revision of the manuscript: AA. Administrative, technical or material support (i.e., requiling and organizing data, constructing databases): EG, BT, ZM, BA, FA. Study supervision: AA.

## Funding

This work was supported by INSERM, ITMO Cancer for its financial support under contract PC201201-084862 “Physiques, mathématiques ou sciences de l'ingénieur appliqués au Cancer,” the Russian Foundation for Basic Research (grant 16-41-590235), the Perm Regional Government (Russia) with the contract “Multiscale approaches in mechanobiology for early cancer diagnosis” and the Maine Cancer Foundation.

### Conflict of interest statement

The authors declare that the research was conducted in the absence of any commercial or financial relationships that could be construed as a potential conflict of interest.
