# Supplementary material for: Comparative Multifractal Analysis of Dynamic Infrared Thermograms and X-Ray Mammograms Enlightens Changes in the Environment of Malignant Tumors
Source: Front Physiol. 2016 Aug 9;7:336. doi: 10.3389/fphys.2016.00336 (PMC4977307; doi:10.3389/fphys.2016.00336)
Supplement: Supplementary file 1 [file Presentation1.PDF]

Supplementary Material: Comparative multifractal analysis of dynamic infrared thermograms and X-ray mammograms enlightens changes in the environment of malignant tumors

E. Gerasimova-Chechkina, B. Toner, Z. Marin, B. Audit, S. G. Roux, F. Argoul, A. Khalil, O. Gileva, O. Naimark and A. Arneodo *

*Correspondence:
Alain Arneodo:
alain.arneodo@ens-lyon.fr

1 SUPPLEMENTARY TABLES AND FIGURES

1.1 Tables

Table S1. Set of (33) analyzed patients with age, cancerous breast (Right or Left), stage, size and depth of the malignant tumor and histology status.

	Age	Breast	Stage	Size (cm)	Depth (cm)	Location	Histological DS
**1**	58	L	IIIa	1.57	4	border outer-inner upper quadrants	invasive ductal cancer
**2**	70	R	IIa	2.8	3	border outer-inner upper quadrants	invasive ductal cancer
**3**	53	R	IIa	2.5 × 2.85	5	upper outer quadrant	invasive ductal cancer
**4**	65	L	IIa	2.6 × 3.3	2	border lower-upper outer quadrant	cystadenocarcinoma
**5**	49	L	IIa	4.15 × 3.45	1	border inner-outer upper quadrants	cystadenocarcinoma
**6**	56	L	III	4.5	4	upper outer quadrant	invasive lobular cancer
**7**	47	R	IIa	2.7 × 2.2	3	border inner-outer upper quadrants	invasive lobular cancer
**8**	82	L	III	1.6 × 1.8	3	upper inner quadrant	invasive ductal cancer
**9**	44	L	IIa	1.8 × 3	6	upper outer quadrant	invasive ductal cancer
**10**	64	R	IIa	-	-		invasive ductal cancer
**11**	55	R	IIb	5.3	1	upper quadrant	ductal cancer
**12**	59	R	Ib	1.8 and 1.26	12	border between upper quadrants	invasive ductal and lobular cancer
**13**	48	L	IIa	1.5	1	underarm area	invasive lobular cancer
**14**	73	R	I	1.2	5	border between upper quadrants	invasive ductal cancer
**15**	81	R	I	-	-		invasive ductal cancer
**16**	41	R	IIa	3.4	7	upper inner quadrant	invasive lobular cancer
**17**	53	R	IIa	3 × 1.5 × 1.4	4	upper outer quadrant	invasive lobular and ductal cancer
**18**	37	L	IIa	3.49 × 2.39	6	upper outer quadrant	invasive ductal cancer
**19**	63	L	-	-	-		invasive ductal cancer
**20**	56	R	IIa	3.1 × 2.5 × 3.6	3	upper outer quadrant	invasive lobular and ductal cancer
**21**	45	R	IIIb	6.5 × 4	2	upper outer qadrant	invasive cancer
**22**	74	R	IIa	-	-		invasive ductal cancer
**23**	40	L	-	2.9 × 3.4	1	border between upper quadrants	invasive lobular cancer
**24**	49	L	IIa	1.7 × 2.2	2	border between outer quadrants	Paget disease of the nipple
**25**	41	L	IIa	2.5 and 1.8 × 2, 2.7 × 1.7	3	border between upper quadrants, upper outer quadrant	invasive ductal cancer
**26**	83	R	Susp. ms	1.9	1	underarm area	no metastasis
**27**	62	L	IIa	2.2 × 2.1	2	border between inner quadrants	invasive ductal and lobular cancer
**28**	76	L	IIb	3.49	8	border between lower quadrants	invasive ductal cancer
**29**	57	R	II	2.1 × 1.6	6	border between lower quadrants	invasive ductal cancer
**30**	54	R	IIa	3.5	3	upper outer quadrant	invasive ductal cancer
**31**	56	R	IIb	-	-		invasive ductal cancer
**32**	55	R	IIb	4.4 × 3 × 2.5	9	border between upper quadrants	invasive ductal cancer
**33**	37	R	IIa	1.57	1.5	border upper-lower outer quadrants, close to the nipple	invasive lobular cancer

Table S2. Results of the 2D WTMM multifractal analysis of the CC and MLO mammographic views of the two breasts of our patients with breast cancer. Number (and percentage) of blue N_b ($H < 0.45$), yellow N_y ($0.45 \leq H \leq 0.55$), red N_r ($H > 0.55$) and pink N_n (no scaling) squares and total number of squares.

	Cancerous breast					Contralateral unaffected breast				
	N_b	N_y	N_r	N_n	**Total**	N_b	N_y	N_r	N_n	**Total**
**1-CC**	54 (68.3%)	10 (12.7%)	6 (7.6%)	9 (11.4%)	79 (100 %)	54 (60.7%)	11 (12.4%)	7 (7.9%)	17 (19.1%)	89 (100 %)
**1-MLO**	62 (70.5%)	8 (9.1%)	11 (12.5%)	7 (8.0%)	88 (100 %)	70 (70.7%)	12 (12.1%)	5 (5.1%)	12 (12.1%)	99 (100 %)
**2-CC**	39 (83.0%)	7 (14.9%)	0 (0.0%)	1 (2.1%)	47 (100 %)	50 (94.3%)	2 (3.8%)	1 (1.9%)	0 (0.0%)	53 (100 %)
**2-MLO**	51 (85.0%)	9 (15.0%)	0 (0.0%)	0 (0.0%)	60 (100 %)	54 (91.5%)	4 (6.8%)	1 (1.7%)	0 (0.0%)	59 (100 %)
**3-CC**	57 (70.4%)	13 (16.1%)	6 (7.4%)	5 (6.2%)	81 (100 %)	54 (62.1%)	24 (27.6%)	5 (5.7%)	4 (4.6%)	87 (100 %)
**3-MLO**	63 (72.4%)	15 (17.2%)	6 (6.9%)	3 (3.4%)	87 (100 %)	78 (78.0%)	12 (12.0%)	5 (5.0%)	5 (5.0%)	100 (100 %)
**4-CC**	34 (63.0%)	14 (25.9%)	4 (7.4%)	2 (3.7%)	54 (100 %)	33 (61.1%)	12 (22.2%)	6 (11.1%)	3 (5.6%)	54 (100 %)
**4-MLO**	42 (68.8%)	15 (24.6%)	3 (4.9%)	1 (1.6%)	61 (100 %)	44 (71.0%)	10 (16.1%)	5 (8.1%)	3 (4.8%)	62 (100 %)
**5-CC**	69 (70.4%)	8 (8.2%)	5 (5.1%)	16 (16.3%)	98 (100 %)	70 (70.0%)	10 (10.0%)	13 (13.0%)	7 (7.0%)	100 (100 %)
**5-MLO**	61 (65.6%)	19 (20.4%)	1 (1.1%)	12 (12.9%)	93 (100 %)	80 (87.0%)	2 (2.2%)	5 (5.4%)	5 (5.4%)	92 (100 %)
**6-CC**	61 (57.0%)	24 (22.4%)	15 (14.0%)	7 (6.5%)	107 (100 %)					
**6-MLO**	111 (72.1%)	19 (12.3%)	6 (3.9%)	18 (11.7%)	154 (100 %)					
**7-CC**	45 (66.2%)	12 (17.6%)	10 (14.7%)	1 (1.5%)	68 (100 %)	29 (41.4%)	13 (18.6%)	18 (25.7%)	10 (14.3%)	70 (100 %)
**7-MLO**	58 (59.2%)	15 (15.3%)	20 (20.4%)	5 (5.1%)	98 (100 %)	40 (53.3%)	11 (14.7%)	18 (24.0%)	6 (8.0%)	75 (100 %)
**8-CC**	166 (82.2%)	19 (9.4%)	4 (2.0%)	13 (6.4%)	202 (100 %)	171 (84.7%)	11 (5.4%)	11 (5.4%)	9 (4.5%)	202 (100 %)
**8-MLO**	175 (86.6%)	17 (8.4%)	4 (2.0%)	6 (3.0%)	202 (100 %)	184 (89.8%)	8 (3.9%)	5 (2.4%)	8 (3.9%)	205 (100 %)
**9-CC**	64 (61.0%)	12 (11.4%)	13 (12.4%)	16 (15.2%)	105 (100 %)	69 (66.3%)	10 (9.6%)	15 (14.4%)	10 (9.6%)	104 (100 %)
**9-MLO**	74 (65.5%)	12 (10.6%)	16 (14.2%)	11 (9.7%)	113 (100 %)	71 (65.7%)	9 (8.3%)	18 (16.7%)	10 (9.3%)	108 (100 %)
**11-CC**	29 (54.7%)	9 (17.0%)	9 (17.0%)	6 (11.3%)	53 (100 %)	37 (90.2%)	3 (7.3%)	0 (0.0%)	1 (2.4%)	41 (100 %)
**11-MLO**	53 (69.7%)	9 (11.8%)	6 (7.9%)	8 (10.5%)	76 (100 %)	61 (96.8%)	1 (1.6%)	0 (0.0%)	1 (1.6%)	63 (100 %)
**12-CC**	119 (82.1%)	15 (10.3%)	2 (1.4%)	9 (6.2%)	145 (100 %)	126 (82.9%)	3 (2.0%)	4 (2.6%)	19 (12.5%)	152 (100 %)
**12-MLO**	124 (80.0%)	15 (9.7%)	4 (2.6%)	12 (7.7%)	155 (100 %)	129 (82.2%)	4 (2.5%)	6 (3.8%)	18 (11.5%)	157 (100 %)
**13-CC**	65 (68.4%)	23 (24.2%)	0 (0.0%)	7 (7.4%)	95 (100 %)	100 (98.0%)	1 (1.0%)	0 (0.0%)	1 (1.0%)	102 (100 %)
**13-MLO**	70 (68.0%)	28 (27.2%)	2 (1.9%)	3 (2.9%)	103 (100 %)	103 (86.5%)	7 (5.9%)	2 (1.7%)	7 (5.9%)	119 (100 %)
**14-CC**	82 (93.2%)	4 (4.5%)	0 (0.0%)	2 (2.3%)	88 (100 %)	98 (96.1%)	1 (1.0%)	0 (0.0%)	3 (2.9%)	102 (100 %)
**14-MLO**	103 (90.3%)	6 (5.3%)	0 (0.0%)	5 (4.4%)	114 (100 %)	111 (94.1%)	4 (3.4%)	0 (0.0%)	3 (2.5%)	118 (100 %)
**16-CC**	96 (84.2%)	10 (8.8%)	0 (0.0%)	8 (7.0%)	114 (100 %)	124 (89.9%)	3 (2.2%)	1 (0.7%)	10 (7.2%)	138 (100 %)
**16-MLO**	132 (85.7%)	17 (11.0%)	0 (0.0%)	5 (3.2%)	154 (100 %)	125 (89.3%)	6 (4.3%)	3 (2.1%)	6 (4.3%)	140 (100 %)
**17-CC**	79 (88.8%)	9 (10.1%)	0 (0.0%)	1 (1.1%)	89 (100 %)	75 (94.9%)	1 (1.3%)	0 (0.0%)	3 (3.8%)	79 (100 %)
**17-MLO**	83 (74.8%)	14 (12.6%)	4 (3.6%)	10 (9.0%)	111 (100 %)	69 (85.2%)	6 (7.4%)	0 (0.0%)	6 (7.4%)	81 (100 %)
**18-CC**	105 (79.5%)	14 (10.6%)	6 (4.5%)	7 (5.3%)	132 (100 %)	129 (96.3%)	2 (1.5%)	0 (0.0%)	3 (2.2%)	134 (100 %)
**18-MLO**	94 (79.0%)	18 (15.1%)	5 (4.2%)	2 (1.7%)	119 (100 %)	139 (91.5%)	2 (1.3%)	6 (3.9%)	5 (3.3%)	152 (100 %)
**19-CC**	94 (89.5%)	10 (9.5%)	0 (0.0%)	1 (1.0%)	105 (100 %)	84 (95.5%)	1 (1.1%)	0 (0.0%)	3 (3.4%)	88 (100 %)
**19-MLO**	114 (93.4%)	6 (4.9%)	0 (0.0%)	2 (1.6%)	122 (100 %)	90 (93.8%)	0 (0.0%)	4 (4.2%)	2 (2.1%)	96 (100 %)
**20-CC**	75 (70.8%)	24 (22.6%)	3 (2.8%)	4 (3.8%)	106 (100 %)	98 (86.7%)	8 (7.1%)	4 (3.5%)	3 (2.7%)	113 (100 %)
**20-MLO**	82 (73.2%)	22 (19.6%)	7 (6.2%)	1 (0.9%)	112 (100 %)	98 (81.7%)	9 (7.5%)	9 (7.5%)	4 (3.3%)	120 (100 %)
**21-CC**	102 (73.9%)	14 (10.1%)	6 (4.3%)	16 (11.6%)	138 (100 %)	119 (93.0%)	5 (3.9%)	3 (2.3%)	1 (0.8%)	128 (100 %)
**21-MLO**	113 (80.1%)	12 (8.5%)	5 (3.5%)	11 (7.8%)	141 (100 %)	130 (90.3%)	5 (3.5%)	6 (4.2%)	3 (2.1%)	144 (100 %)
**23-CC**	26 (65.0%)	5 (12.5%)	6 (15.0%)	3 (7.5%)	40 (100 %)	23 (62.2%)	9 (24.3%)	4 (10.8%)	1 (2.7%)	37 (100 %)
**23-MLO**	36 (55.4%)	11 (16.9%)	13 (20.0%)	5 (7.7%)	65 (100 %)	52 (76.5%)	7 (10.3%)	9 (13.2%)	0 (0.0%)	68 (100 %)
**24-CC**	26 (44.8%)	24 (41.4%)	7 (12.1%)	1 (1.7%)	58 (100 %)	45 (75.0%)	7 (11.7%)	7 (11.7%)	1 (1.7%)	60 (100 %)
**24-MLO**	37 (60.7%)	15 (24.6%)	7 (11.5%)	2 (3.3%)	61 (100 %)	61 (80.3%)	9 (11.8%)	4 (5.3%)	2 (2.6%)	76 (100 %)
**25-CC**	49 (59.0%)	19 (22.9%)	2 (2.4%)	13 (15.7%)	83 (100 %)	66 (84.6%)	5 (6.4%)	4 (5.1%)	3 (3.8%)	78 (100 %)
**25-MLO**	46 (56.8%)	17 (21.0%)	4 (4.9%)	14 (17.3%)	81 (100 %)	56 (63.6%)	9 (10.2%)	15 (17.1%)	8 (9.1%)	88 (100 %)
**26-CC**	29 (87.9%)	4 (12.1%)	0 (0.0%)	0 (0.0%)	33 (100 %)	77 (86.5%)	7 (7.9%)	1 (1.1%)	4 (4.5%)	89 (100 %)
**26-MLO**	78 (85.7%)	10 (11.0%)	2 (2.2%)	1 (1.1%)	91 (100 %)	93 (93.0%)	6 (6.0%)	0 (0.0%)	1 (1.0%)	100 (100 %)
**27-CC**	140 (87.5%)	13 (8.1%)	6 (3.8%)	1 (0.6%)	160 (100 %)	160 (98.2%)	1 (0.6%)	2 (1.2%)	0 (0.0%)	163 (100 %)
**27-MLO**	136 (90.1%)	11 (7.3%)	2 (1.3%)	2 (1.3%)	151 (100 %)	167 (92.3%)	4 (2.2%)	3 (1.7%)	7 (3.9%)	181 (100 %)
**28-CC**	105 (84.0%)	13 (10.4%)	1 (0.8%)	6 (4.8%)	125 (100 %)	133 (96.4%)	4 (2.9%)	1 (0.7%)	0 (0.0%)	138 (100 %)
**28-MLO**	87 (79.8%)	16 (14.7%)	4 (3.7%)	2 (1.8%)	109 (100 %)	121 (93.1%)	6 (4.6%)	2 (1.5%)	1 (0.8%)	130 (100 %)
**29-CC**	65 (76.5%)	14 (16.5%)	4 (4.7%)	2 (2.4%)	85 (100 %)	76 (81.7%)	9 (9.7%)	3 (3.2%)	5 (5.4%)	93 (100 %)
**29-MLO**	73 (80.2%)	14 (15.4%)	4 (4.4%)	0 (0.0%)	91 (100 %)	87 (90.6%)	5 (5.2%)	2 (2.1%)	2 (2.1%)	96 (100 %)
**30-CC**	66 (76.7%)	8 (9.3%)	4 (4.7%)	8 (9.3%)	86 (100 %)	66 (78.6%)	12 (14.3%)	3 (3.6%)	3 (3.6%)	84 (100 %)
**30-MLO**	62 (65.3%)	13 (13.7%)	13 (13.7%)	7 (7.4%)	95 (100 %)	72 (75.0%)	9 (9.4%)	5 (5.2%)	10 (10.4%)	96 (100 %)
**31-CC**	33 (62.3%)	12 (22.6%)	6 (11.3%)	2 (3.8%)	53 (100 %)	46 (88.5%)	3 (5.8%)	2 (3.8%)	1 (1.9%)	52 (100 %)
**31-MLO**	46 (62.2%)	19 (25.7%)	7 (9.5%)	2 (2.7%)	74 (100 %)	55 (75.3%)	7 (9.6%)	6 (8.2%)	5 (6.8%)	73 (100 %)
**32-CC**	54 (37.8%)	40 (28.0%)	20 (14.0%)	29 (20.3%)	143 (100 %)	80 (47.6%)	30 (17.9%)	34 (20.2%)	24 (14.3%)	168 (100 %)
**32-MLO**	73 (48.3%)	36 (23.8%)	18 (11.9%)	24 (15.9%)	151 (100 %)	105 (64.8%)	19 (11.7%)	17 (10.5%)	21 (13.0%)	162 (100 %)
**33-CC**	10 (40.0%)	8 (32.0%)	6 (24.0%)	1 (4.0%)	25 (100 %)	14 (29.8%)	4 (8.5%)	15 (31.9%)	14 (29.8%)	47 (100 %)
**33-MLO**	16 (48.5%)	10 (30.3%)	5 (15.2%)	2 (6.1%)	33 (100 %)	10 (20.8%)	8 (16.7%)	14 (29.2%)	16 (33.3%)	48 (100 %)

Table S3. Statistics of monofractal uncorrelated $H = 0.5$ yellow square clusters in the CC and MLO mammographic views of the two breasts of our patients with breast cancer. N_y = number of $H = 0.5$ squares; $N_{cluster}$ = number of $H = 0.5$ square clusters; $Size_{cluster}$ = number of $H = 0.5$ squares in each cluster. Clusters are defined by squares sharing a common edge.

	Cancerous breast			Contralateral unaffected breast		
	**Total N_y**	**$N_{cluster}$**	**$Size_{cluster}$**	**Total N_y**	**$N_{cluster}$**	**$Size_{cluster}$**
**1-CC**	10	5	4 3 1 1 1	11	11	1 1 1 1 1 1 1 1 1 1 1
**1-MLO**	8	6	3 1 1 1 1 1	12	5	4 4 2 1 1
**2-CC**	7	3	5 1 1	2	2	1 1
**2-MLO**	9	2	8 1	4	3	2 1 1
**3-CC**	13	3	11 1 1	24	5	17 4 1 1 1
**3-MLO**	15	5	9 2 2 1 1	12	10	2 2 1 1 1 1 1 1 1 1
**4-CC**	14	5	5 4 2 2 1	12	8	3 2 2 1 1 1 1 1
**4-MLO**	15	6	10 1 1 1 1 1	10	7	4 1 1 1 1 1 1
**5-CC**	8	5	3 2 1 1 1	10	6	4 2 1 1 1 1
**5-MLO**	19	9	4 4 3 2 2 1 1 1 1	2	2	1 1
**6-CC**	24	8	11 3 3 2 2 1 1 1	0	0	0
**6-MLO**	19	6	11 4 1 1 1 1	0	0	0
**7-CC**	12	4	9 1 1 1	13	5	6 3 2 1 1
**7-MLO**	15	5	10 2 1 1 1	11	8	3 2 1 1 1 1 1 1
**8-CC**	19	9	6 4 2 2 1 1 1 1 1	11	3	8 2 1
**8-MLO**	17	3	14 2 1	8	6	2 2 1 1 1 1
**9-CC**	12	11	2 1 1 1 1 1 1 1 1 1 1	10	6	3 2 2 1 1 1
**9-MLO**	12	5	6 2 2 1 1	9	8	2 1 1 1 1 1 1 1
**11-CC**	9	4	5 2 1 1	3	2	2 1
**11-MLO**	9	8	2 1 1 1 1 1 1 1	1	1	1
**12-CC**	15	7	5 3 3 1 1 1 1	3	3	1 1 1
**12-MLO**	15	8	4 4 2 1 1 1 1 1	4	4	1 1 1 1
**13-CC**	23	6	15 3 2 1 1 1	1	1	1
**13-MLO**	28	3	26 1 1	7	6	2 1 1 1 1 1
**14-CC**	4	2	3 1	1	1	1
**14-MLO**	6	3	2 2 2	4	1	4
**16-CC**	10	5	5 2 1 1 1	3	2	2 1
**16-MLO**	17	4	13 2 1 1	6	4	2 2 1 1
**17-CC**	9	4	6 1 1 1	1	1	1
**17-MLO**	14	6	8 2 1 1 1 1	6	3	3 2 1
**18-CC**	14	9	4 3 1 1 1 1 1 1 1	2	2	1 1
**18-MLO**	18	10	4 3 2 2 2 1 1 1 1 1	2	2	1 1
**19-CC**	10	2	8 2	1	1	1
**19-MLO**	6	2	4 2	0	0	0
**20-CC**	24	4	17 4 2 1	8	4	4 2 1 1
**20-MLO**	22	7	7 7 3 2 1 1 1	9	6	3 2 1 1 1 1
**21-CC**	14	10	4 2 1 1 1 1 1 1 1 1	5	4	2 1 1 1
**21-MLO**	12	10	2 2 1 1 1 1 1 1 1 1	5	5	1 1 1 1 1
**23-CC**	5	3	3 1 1	9	4	5 2 1 1
**23-MLO**	11	6	4 2 2 1 1 1	7	4	4 1 1 1
**24-CC**	24	6	12 5 4 1 1 1	7	4	3 2 1 1
**24-MLO**	15	7	9 1 1 1 1 1 1	9	6	3 2 1 1 1 1
**25-CC**	19	6	9 4 3 1 1 1	5	4	2 1 1 1
**25-MLO**	17	6	5 4 4 2 1 1	9	5	3 3 1 1 1
**26-CC**	4	2	2 2	7	6	2 1 1 1 1 1
**26-MLO**	10	2	8 2	6	2	4 2
**27-CC**	13	5	8 2 1 1 1	1	1	1
**27-MLO**	11	6	4 3 1 1 1 1	4	3	2 1 1
**28-CC**	13	8	4 2 2 1 1 1 1 1	4	3	2 1 1
**28-MLO**	16	7	4 4 4 1 1 1 1	6	5	2 1 1 1 1
**29-CC**	14	5	6 5 1 1 1	9	5	3 2 2 1 1
**29-MLO**	14	3	6 6 2	5	2	3 2
**30-CC**	8	6	2 2 1 1 1 1	12	8	3 2 2 1 1 1 1 1
**30-MLO**	13	6	5 4 1 1 1 1	9	5	4 2 1 1 1
**31-CC**	12	2	9 3	3	2	2 1
**31-MLO**	18	7	7 4 2 2 1 1 1	7	5	3 1 1 1 1
**32-CC**	40	15	8 7 4 4 4 4 1 1 1 1 1 1 1 1 1	30	22	4 3 2 2 2 1 1 1 1 1 1 1 1 1 1 1 1 1 1 1 1 1
**32-MLO**	36	18	6 6 4 4 2 2 1 1 1 1 1 1 1 1 1 1 1 1	19	12	4 4 2 1 1 1 1 1 1 1 1 1
**33-CC**	8	6	2 2 1 1 1 1	4	4	1 1 1 1
**33-MLO**	10	2	7 3	8	5	4 1 1 1 1
**Average**	14.3	5.8	6.8	7.1	4.6	2.8
**St. dev.**	6.9	3.1	4.3	5.5	3.5	2.4

1.2 Figures

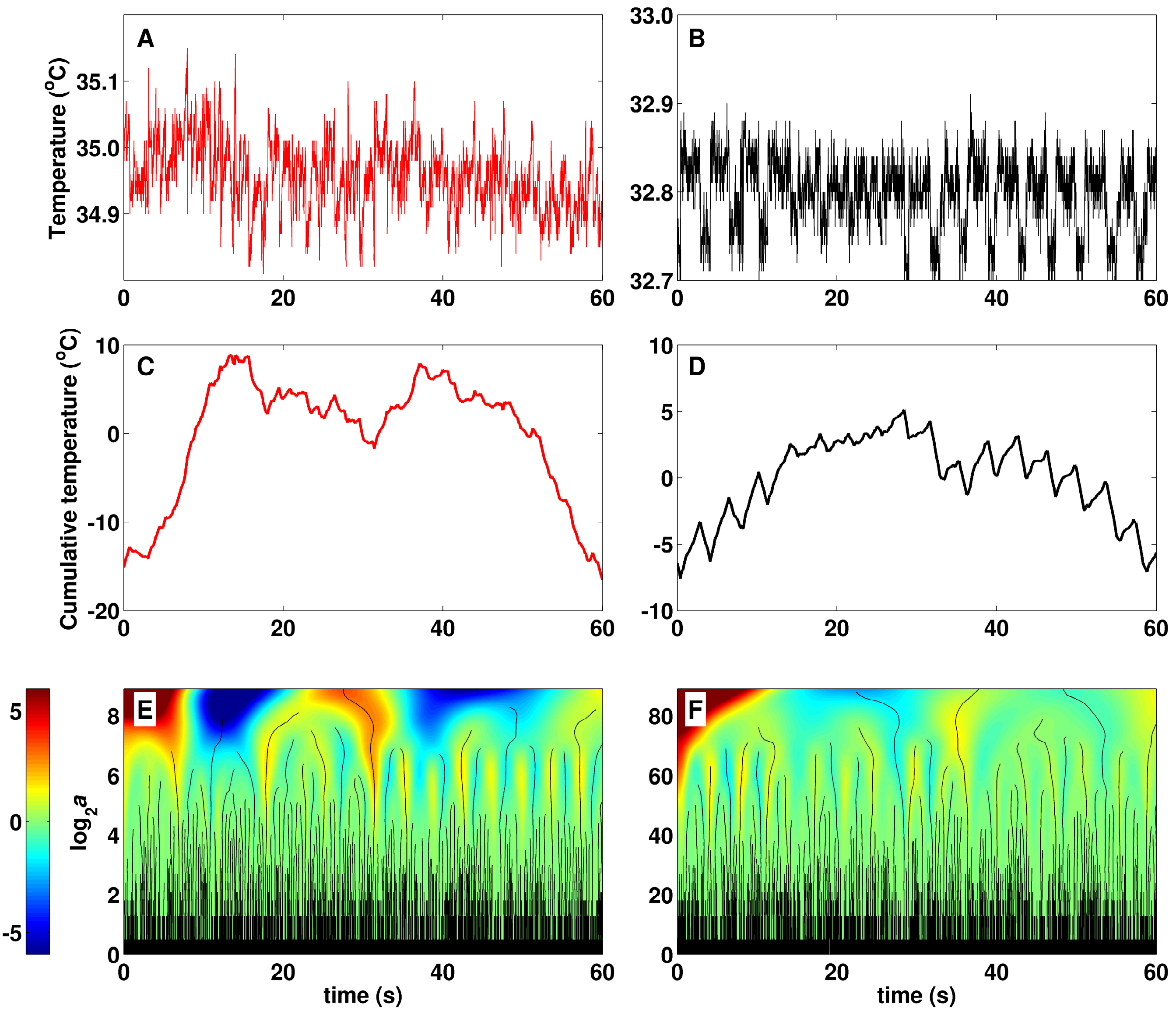

Figure S1. Comparative analysis of the IR thermograms of the cancerous right breast (A, C, E) and contralateral unaffected left breast (B, D, F) of patient 20 (age 56). (A, B) 1 min portion of pixel temperature time-series. (C, D) 1 min portion of cumulative pixel temperature time-series (after removing the overall linear trend). (E, F) 1D WT (Eq. (1)) of the cumulative time-series as coded from black (min $|W(\cdot, a)|$) to red (max $|W(\cdot, a)|$); solid black lines are the WTMM lines that define the WT skeleton. The analazing wavelet is the second-order compactly supported analazing wavelet $\psi_{(3)}^{(2)}$ (see Supplementary Fig. S1 in Ref. (Gerasimova et al., 2014)).

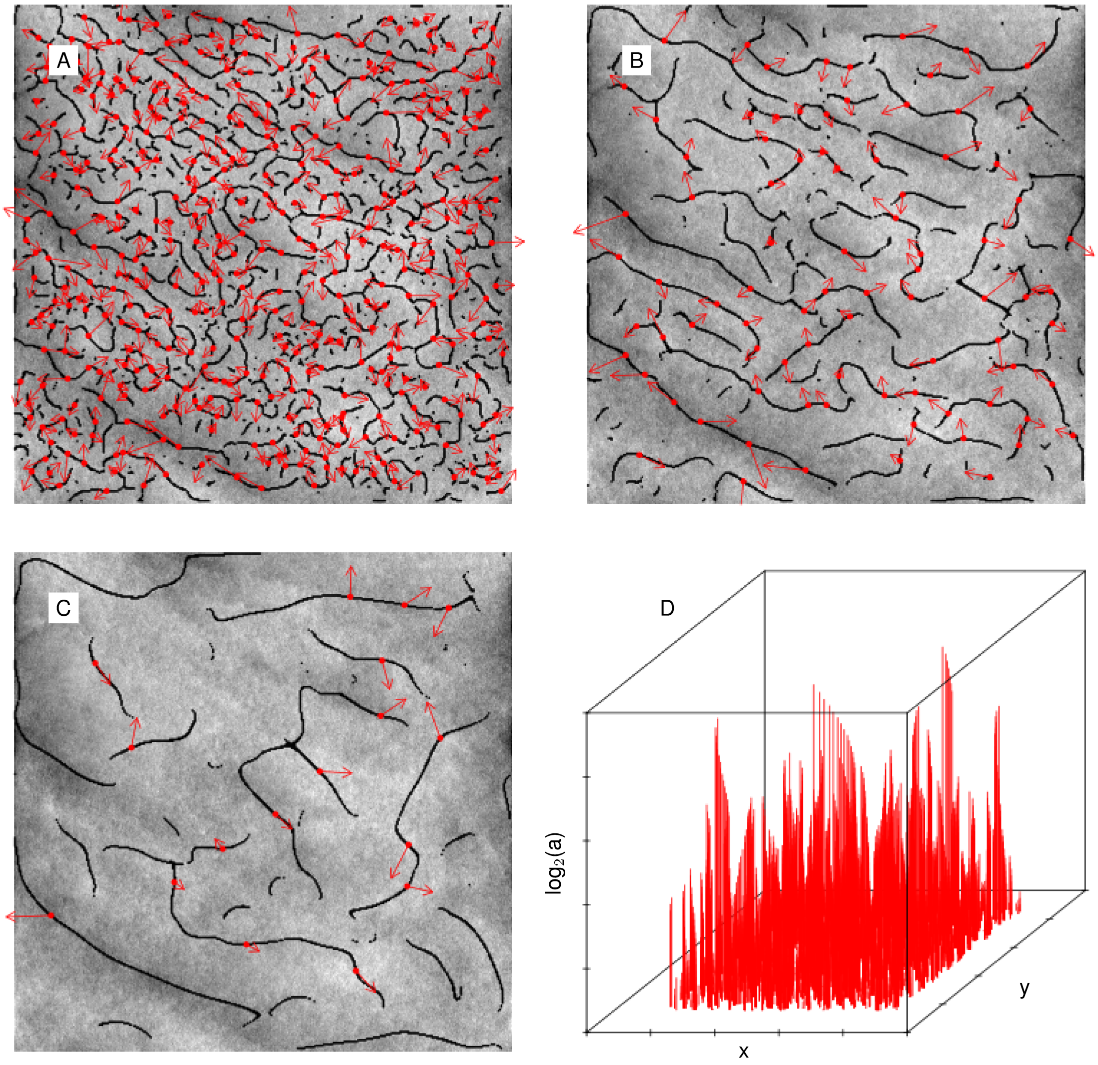

Figure S2. Analysis of the mammograms of patient 20 (age 56) using the 2D WTMM method. In (A) $a = 2^{2.9}\sigma_W$, (B) $a = 2^{1.9}\sigma_W$ and (C) $a = 2^{3.9}\sigma_W$ where ($\sigma_W = 7$ pixels $\simeq$ 0.7 mm), are shown the maxima chains; the local maxima of $\mathcal{M}_\psi$ along these chains are indicated by (•) from which originates an arrow whose length is proportional to $\mathcal{M}_\psi$ (Eq. (4)) and its direction (with respect to x-axis) is given by $\mathcal{A}_\psi$ (Eq. (5)). In (A)–(C) the smoothed image $\phi_{\mathbf{b},a} \star I$ is shown as a grey-scale coded background from white (min) to black (max). The WTMMM are then connected vertically through scales to define the WT skeleton shown in (D). The analyzing wavelets $\psi_1(x,y) = \frac{\partial\phi(x,y)}{\partial x}$ and $\psi_2(x,y) = \frac{\partial\phi(x,y)}{\partial y}$ (Eq. (2)), where $\phi(x,y) = \mathrm{e}^{-(x^2+y^2)/2}$ (Eq. (12)).

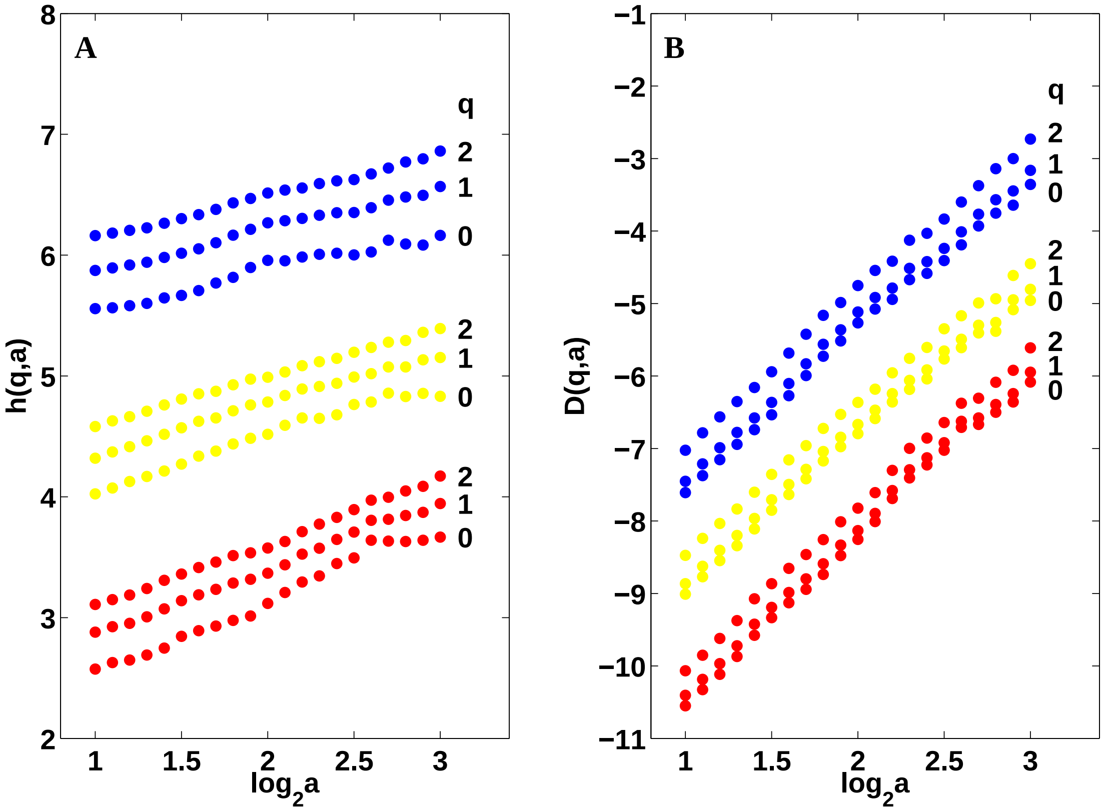

Figure S3. Multifractal analysis of X-ray mammograms of patient 20 (age 56). Comparative analysis of spatial roughness fluctuations in three 256×256 pixel^2 squares in the cancerous right breast with respectively monofractal $H < 0.45$ (blue), $0.45 \leq H \leq 0.55$ (yellow), $H > 0.55$ (red): (A) $h(q, a)$ vs $\ln a$ (Eq.(8)); (B) $D(q, a)$ vs $\ln a$ (Eq.(9)). The $D(h)$ spectra in Fig. 2C were obtained by linear regression fit in (A) and (B) over the range of space-scales $1 \leq \log_2 a \leq 3$.

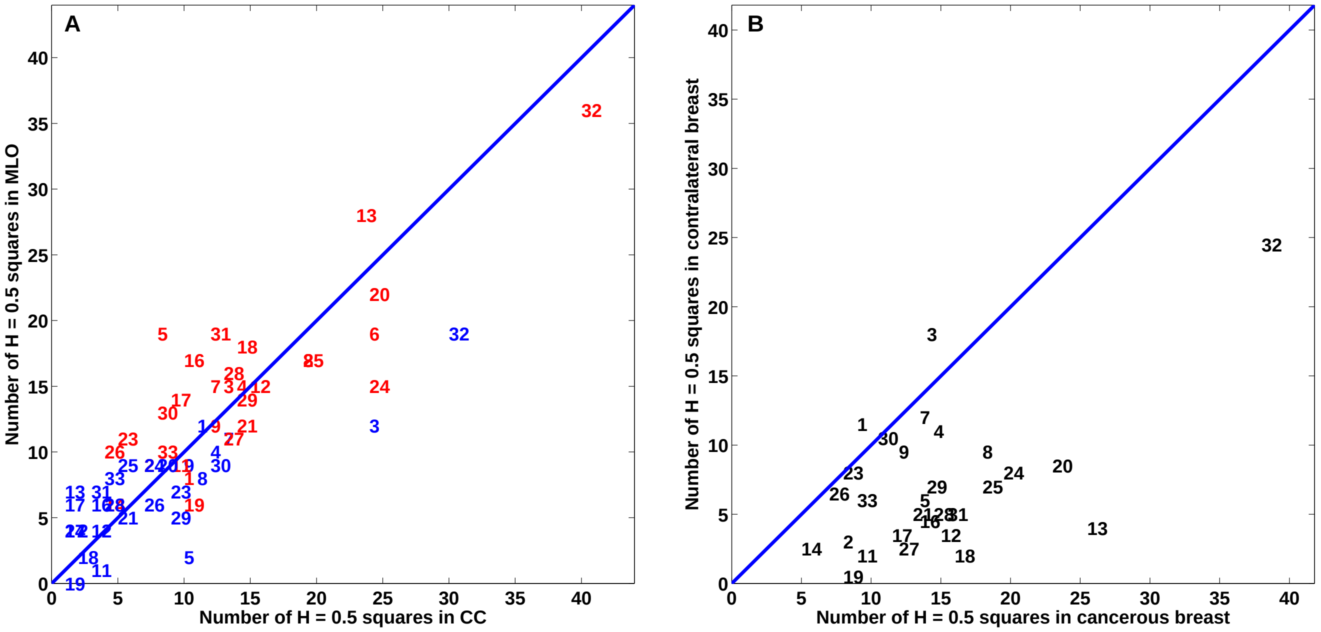

Figure S4. Analysis of the number of monofractal $H = 0.5$ 256×256 pixel^2 squares in the mammograms of the two breasts of 30 patients with breast cancer. (A) Number of $H = 0.5$ squares in cancerous (red) and contralateral unaffected (blue) breasts: MLO view vs. CC view. (B) Number of $H = 0.5$ squares in both MLO and CC views: contralateral unaffected breast vs. cancerous breast. In (A) and (B), the numbers correspond to patient numbers defined in Supplementary Table S1.

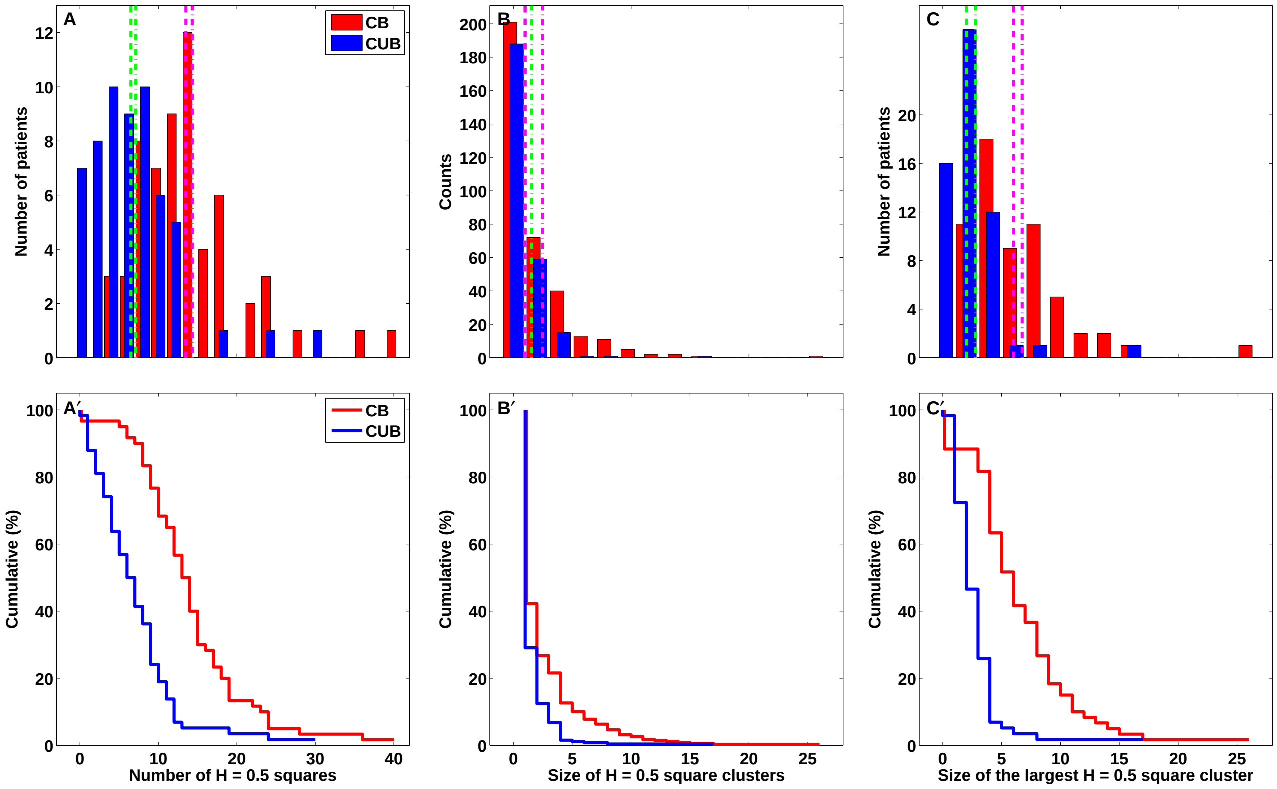

Figure S5. Differential monofractal $H = 0.5$ signature on the (CC and MLO) mammograms of cancerous breasts (CB) and contralateral unaffected breasts (CUB). (A) Histograms of the number of squares in the mammograms of CBs (red) and CUBs (blue) of 30 patients with breast cancer. (B) Histograms of the size of $H = 0.5$ square clusters (see text) in CBs (red) and CUBs (blue). (C) Histograms of the size of the largest $H = 0.5$ square cluster in CBs (red) and CUBs (blue). (A′, B′, C′) same as (A, B, C) for the corresponding cumulative distribution functions. Clusters are defined by squares sharing a common edge. In (A,B,C), the pink and green vertical lines correspond to the mean (dashed line) and median (dashed-dotted line) of the histogram obtained for CBs and CUBs respectively.

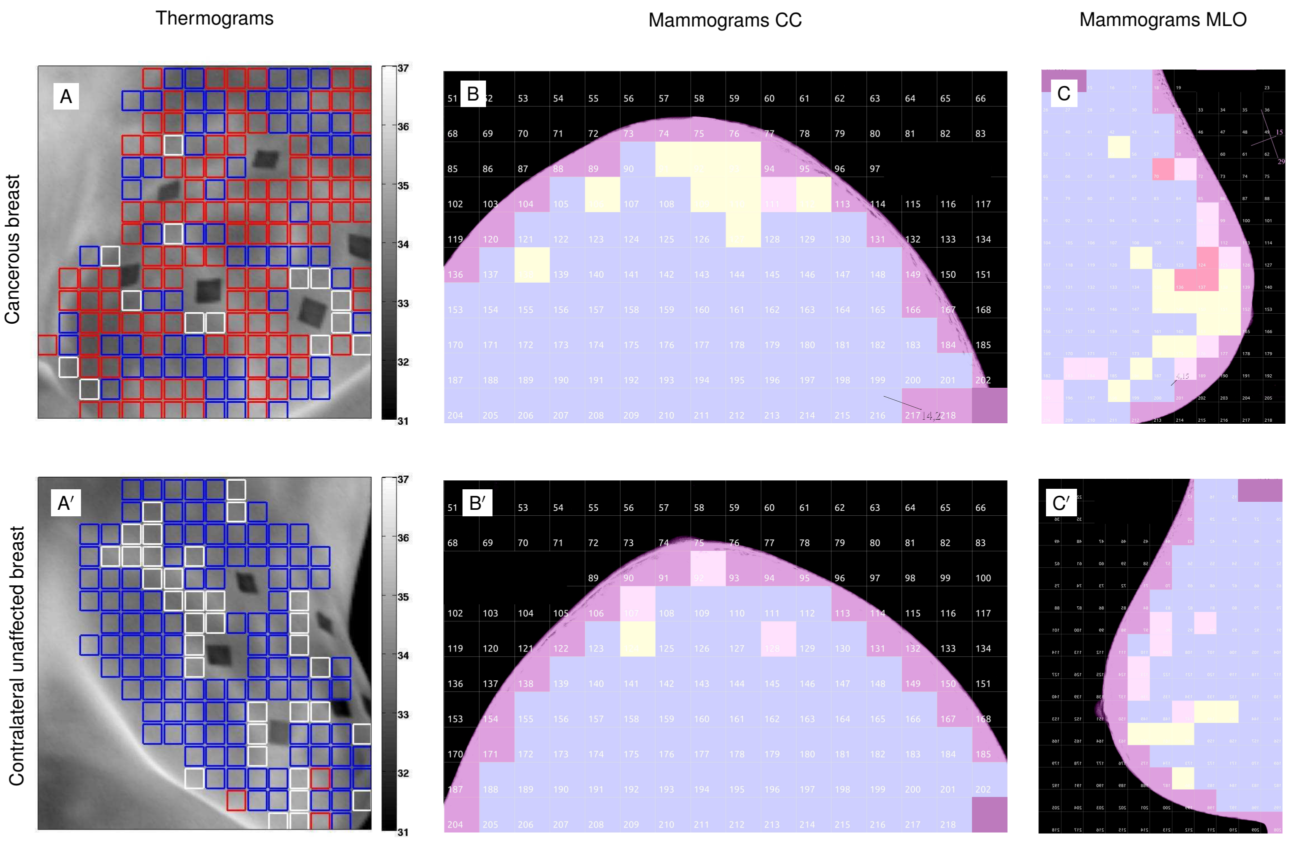

Figure S6. Wavelet-based multifractal segmentation of dynamic infrared thermograms and X-ray mammograms. Patient 17 (age 53): cancerous right breast (A, B, C) and contralateral unaffected left breast (A′, B′, C′). (A, A′) As estimated from the $\tau(q)$ spectrum of skin temperature temporal fluctuations computed with the 1D WTMM method, 8×8 pixel^2 squares spanning 10×10 mm^2 were color coded according to monofractal ($c_2 < 0.03$, red), multifractal ($c_2 \geq 0.03$, blue) and no scaling (white) diagnostic, where c_2 is the intermittency coefficient that defines the width of the $D(h)$ singularity spectrum (Eqs. (10) and (11)) (Gerasimova et al., 2014). (B, B′) As estimated from the $\tau(q)$ spectra of CC mammographic view computed with the 2D WTMM method, 256×256 pixel^2 squares spanning 12.8×12.8 mm^2 were color coded according to monofractal $H < 0.45$ (blue), $0.45 \leq H \leq 0.55$ (yellow), $H > 0.55$ (red) and no scaling (pink). (C, C′) Same as (B, B′) for MLO mammographic view.

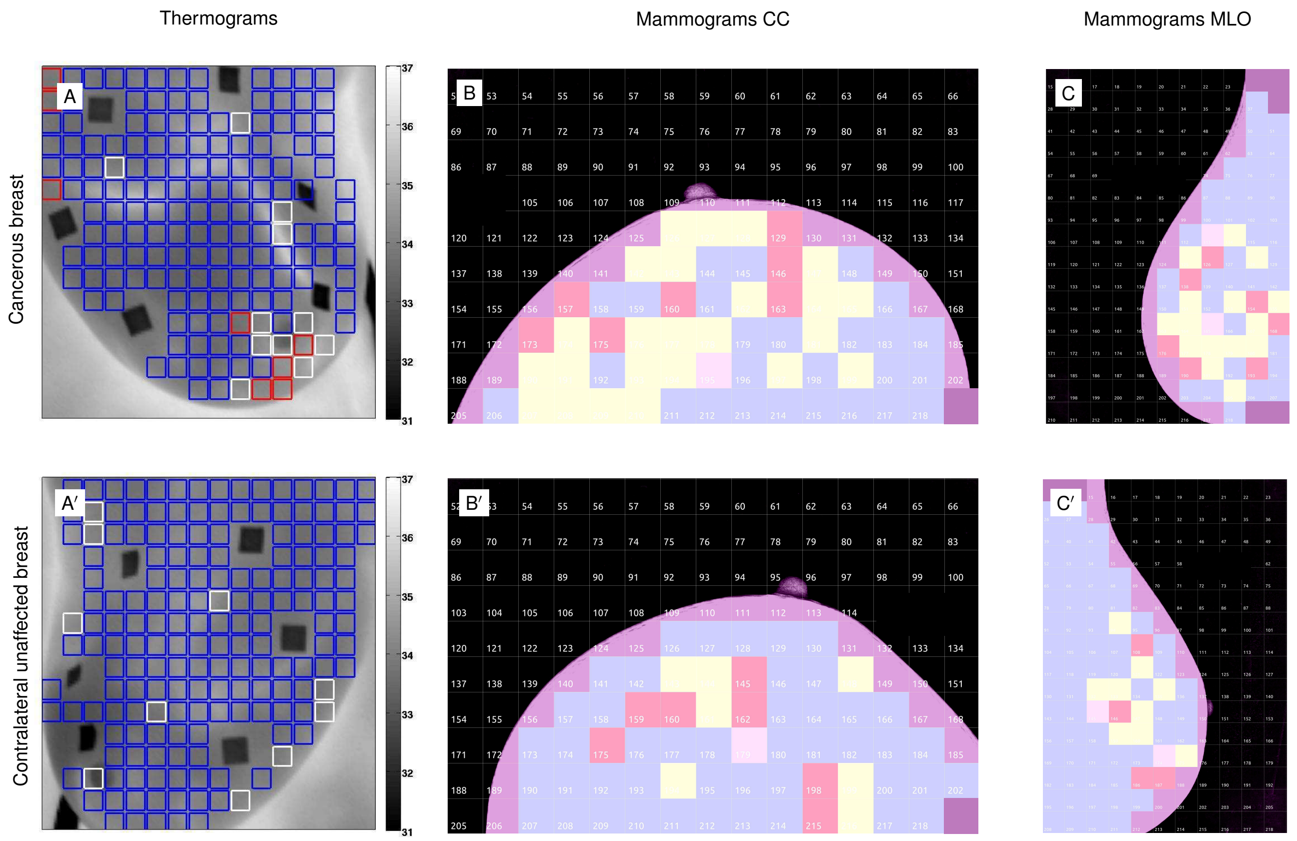

Figure S7. Wavelet-based multifractal segmentation of dynamic infrared thermograms and X-ray mammograms. Patient 24 (age 49): cancerous left breast (A, B, C) and contralateral unaffected right breast (A′, B′, C′). (A, A′) As estimated from the $\tau(q)$ spectrum of skin temperature temporal fluctuations computed with the 1D WTMM method, 8×8 pixel^2 squares spanning 10×10 mm^2 were color coded according to monofractal ($c_2 < 0.03$, red), multifractal ($c_2 \geq 0.03$, blue) and no scaling (white) diagnostic, where c_2 is the intermittency coefficient that defines the width of the $D(h)$ singularity spectrum (Eqs. (10) and (11)) (Gerasimova et al., 2014). (B, B′) As estimated from the $\tau(q)$ spectra of CC mammographic view computed with the 2D WTMM method, 256×256 pixel^2 squares spanning 12.8×12.8 mm^2 were color coded according to monofractal $H < 0.45$ (blue), $0.45 \leq H \leq 0.55$ (yellow), $H > 0.55$ (red) and no scaling (pink). (C, C′) Same as (B, B′) for MLO mammographic view.

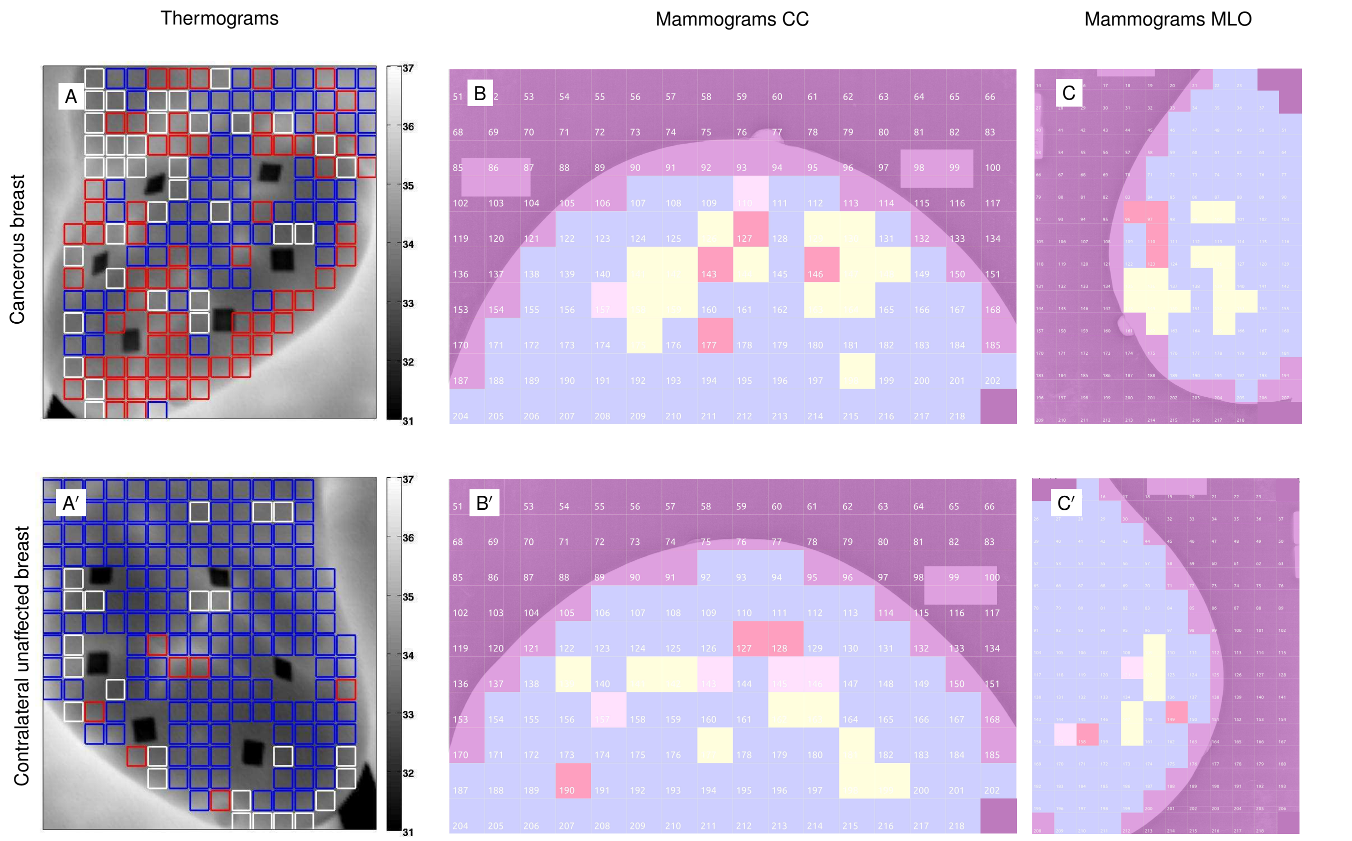

Figure S8. Wavelet-based multifractal segmentation of dynamic infrared thermograms and X-ray mammograms. Patient 29 (age 57): cancerous right breast (A, B, C) and contralateral unaffected left breast (A′, B′, C′). (A, A′) As estimated from the $\tau(q)$ spectrum of skin temperature temporal fluctuations computed with the 1D WTMM method, 8×8 pixel^2 squares spanning 10×10 mm^2 were color coded according to monofractal ($c_2 < 0.03$, red), multifractal ($c_2 \geq 0.03$, blue) and no scaling (white) diagnostic, where c_2 is the intermittency coefficient that defines the width of the $D(h)$ singularity spectrum (Eqs. (10) and (11)) (Gerasimova et al., 2014). (B, B′) As estimated from the $\tau(q)$ spectra of CC mammographic view computed with the 2D WTMM method, 256×256 pixel^2 squares spanning 12.8×12.8 mm^2 were color coded according to monofractal $H < 0.45$ (blue), $0.45 \leq H \leq 0.55$ (yellow), $H > 0.55$ (red) and no scaling (pink). (C, C′) Same as (B, B′) for MLO mammographic view.

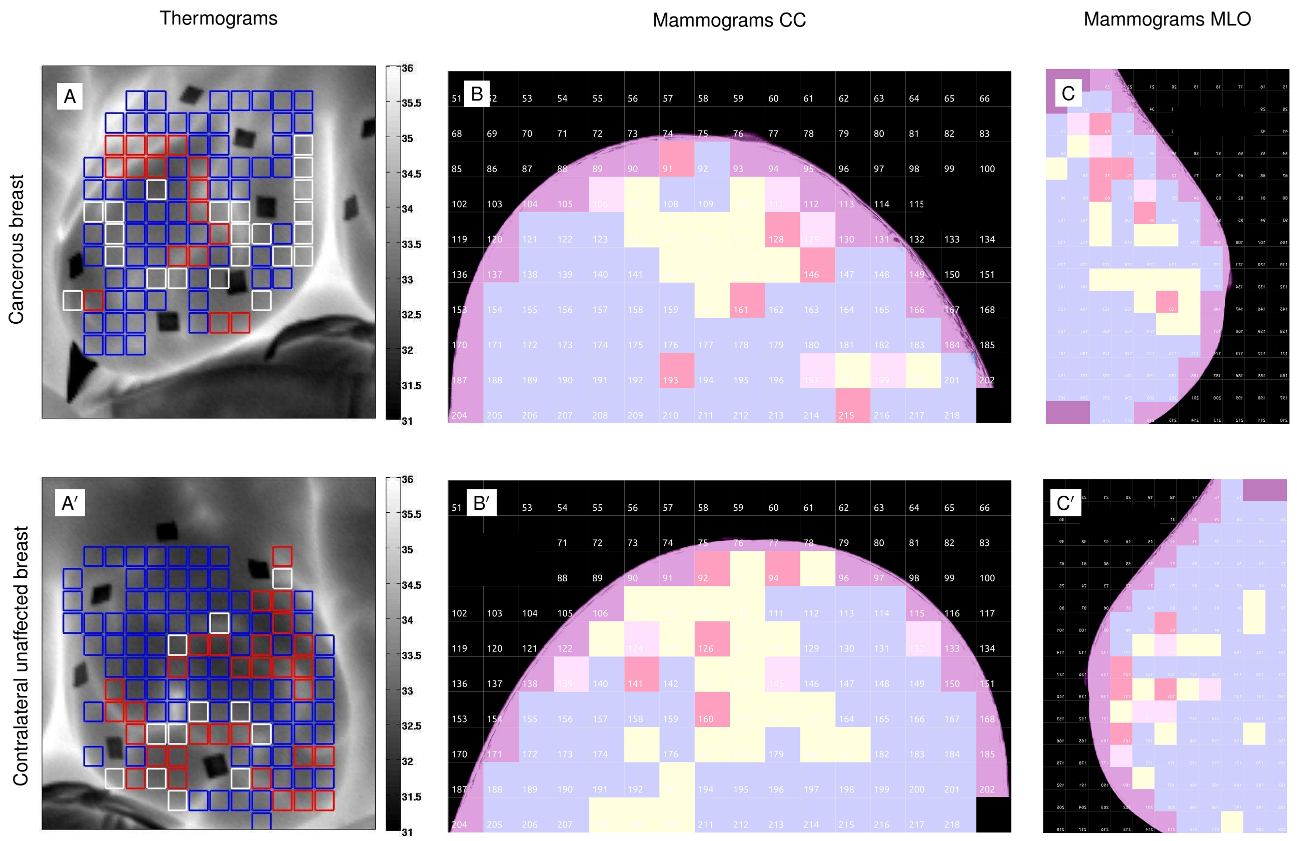

Figure S9. Wavelet-based multifractal segmentation of dynamic infrared thermograms and X-ray mammograms. Patient 3 (age 53): cancerous right breast (A, B, C) and contralateral unaffected left breast (A′, B′, C′). (A, A′) As estimated from the $\tau(q)$ spectrum of skin temperature temporal fluctuations computed with the 1D WTMM method, 8×8 pixel^2 squares spanning 10×10 mm^2 were color coded according to monofractal ($c_2 < 0.03$, red), multifractal ($c_2 \geq 0.03$, blue) and no scaling (white) diagnostic, where c_2 is the intermittency coefficient that defines the width of the $D(h)$ singularity spectrum (Eqs. (10) and (11)) (Gerasimova et al., 2014). (B, B′) As estimated from the $\tau(q)$ spectra of CC mammographic view computed with the 2D WTMM method, 256×256 pixel^2 squares spanning 12.8×12.8 mm^2 were color coded according to monofractal $H < 0.45$ (blue), $0.45 \leq H \leq 0.55$ (yellow), $H > 0.55$ (red) and no scaling (pink). (C, C′) Same as (B, B′) for MLO mammographic view.

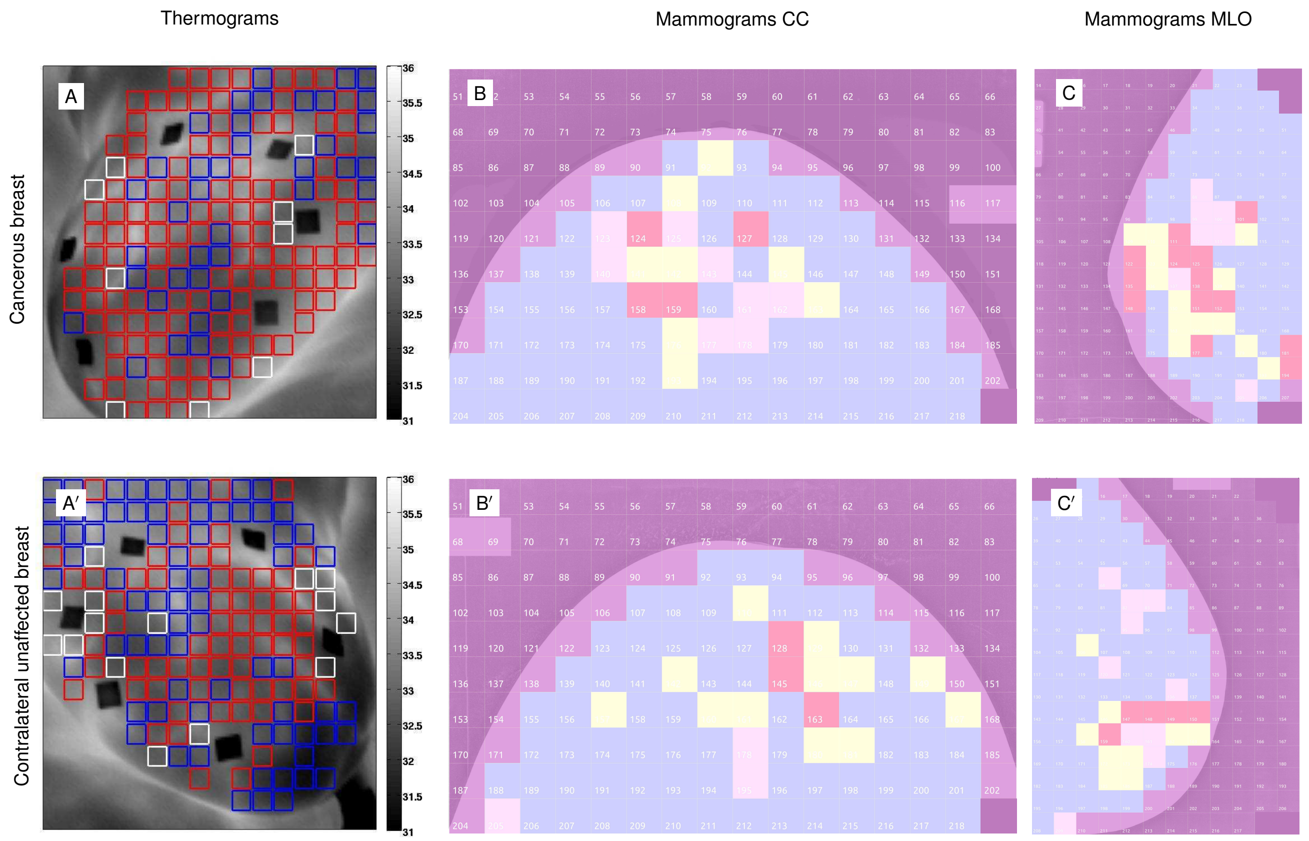

Figure S10. Wavelet-based multifractal segmentation of dynamic infrared thermograms and X-ray mammograms. Patient 30 (age 54): cancerous right breast (A, B, C) and contralateral unaffected left breast (A′, B′, C′). (A, A′) As estimated from the $\tau(q)$ spectrum of skin temperature temporal fluctuations computed with the 1D WTMM method, 8×8 pixel^2 squares spanning 10×10 mm^2 were color coded according to monofractal ($c_2 < 0.03$, red), multifractal ($c_2 \geq 0.03$, blue) and no scaling (white) diagnostic, where c_2 is the intermittency coefficient that defines the width of the $D(h)$ singularity spectrum (Eqs. (10) and (11)) (Gerasimova et al., 2014). (B, B′) As estimated from the $\tau(q)$ spectra of CC mammographic view computed with the 2D WTMM method, 256×256 pixel^2 squares spanning 12.8×12.8 mm^2 were color coded according to monofractal $H < 0.45$ (blue), $0.45 \leq H \leq 0.55$ (yellow), $H > 0.55$ (red) and no scaling (pink). (C, C′) Same as (B, B′) for MLO mammographic view.

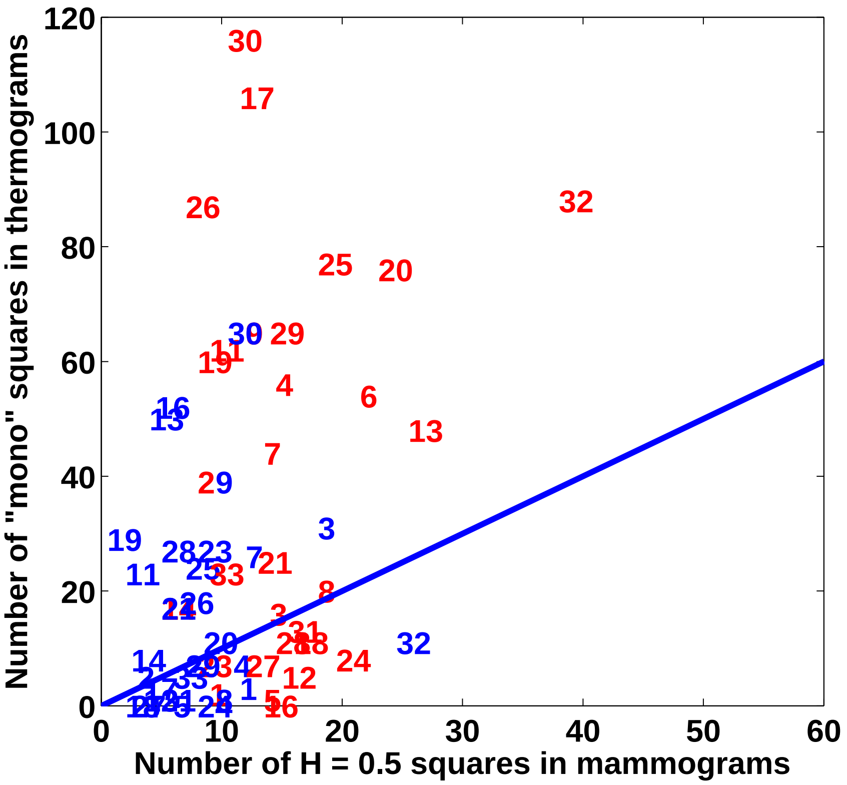

Figure S11. Comparative analysis thermograms and (CC and MLO averaged) mammograms. Number of monofractal squares in thermograms vs. number of $H = 0.5$ squares in mammograms of the cancerous (red) and contralateral unaffected (blue) breasts of the 30 patients with breast cancer. Patient numbers are defined in Supplementary Table S1.
